# Msc1 is a nuclear envelope protein that reinforces DNA repair in late mitosis

**DOI:** 10.1016/j.isci.2024.110250

**Published:** 2024-06-11

**Authors:** Sara Medina-Suárez, Jessel Ayra-Plasencia, Lara Pérez-Martínez, Falk Butter, Félix Machín

**Affiliations:** 1 Unidad de Investigación, Hospital Universitario Nuestra Señora de Candelaria, 38010 Santa Cruz de Tenerife, Spain; 2 Instituto de Tecnologías Biomédicas, Universidad de La Laguna, 38200 San Cristóbal de La Laguna, Spain; 3 Institute of Molecular Biology (IMB), 55128 Mainz, Germany; 4 Institute of Molecular Virology and Cell Biology, Friedrich Loeffler Institute, 17493 Greifswald, Germany; 5 Facultad de Ciencias de la Salud, Universidad Fernando Pessoa Canarias, 35450 Las Palmas de Gran Canaria, Spain

**Keywords:** Biological sciences, Molecular biology, Cell biology

## Abstract

Precise double-strand break (DSB) repair is a paramount for genome stability. Homologous recombination (HR) repairs DSBs when cyclin-dependent kinase (CDK) activity is high, which correlates with the availability of the sister chromatid as a template. However, anaphase and telophase are paradoxical scenarios since high CDK favors HR despite sister chromatids being no longer aligned. To identify factors specifically involved in DSB repair in late mitosis, we have undertaken comparative proteomics in *Saccharomyces cerevisiae* and found that meiotic sister chromatid 1 (Msc1), a poorly characterized nuclear envelope protein, is significantly enriched upon both random and guided DSBs. We further show that Δ*msc1* is more sensitive to DSBs in late mitosis, and has a delayed repair of DBSs, as indicated by increased Rad53 hyperphosphorylation, a higher presence of RPA foci, fewer Rad52 repair factories, and slower HR completion. We propose that Msc1 favors the later stages of HR and the timely completion of DSB repair before cytokinesis.

## Highlights

• Proteomics identifies Msc1 as upregulated after DNA damage in late mitosis
• Lack of Msc1 causes low tolerance to DNA damage and checkpoint hyperactivation
• Msc1 forms nuclear envelope patches associated with presence of DNA repair foci
• Msc1 accelerates homologous recombination downstream of DNA end resection

## Graphical abstract

**Figure:**
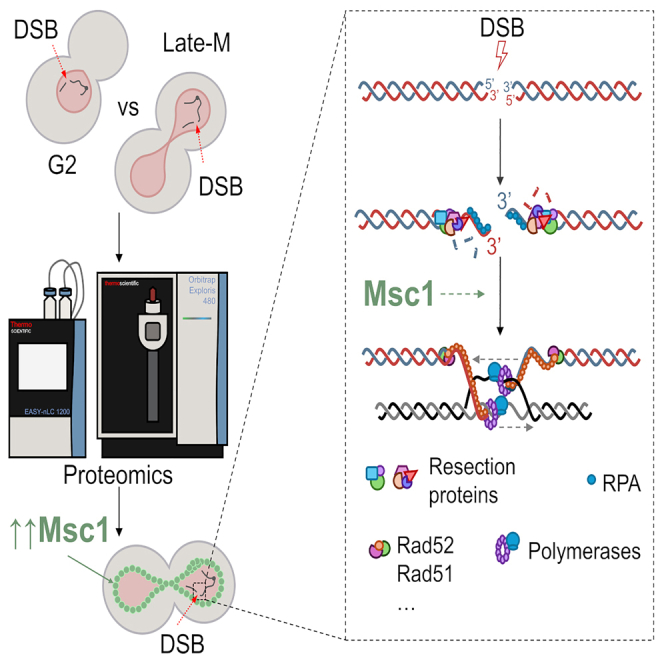

## Introduction

DNA double-strand breaks (DSBs) pose a threat for cell survival and genome stability, playing a major role in carcinogenesis.^1^^,^^2^^,^^3^ Cells deal with DSBs through two main DNA repair mechanisms, non-homologous end-joining (NHEJ) and homologous recombination (HR). The former comprises error-prone pathways that join two broken DNA ends, whereas the latter involves pathways that use intact homologous sequences to restore the broken DNA sequence.^2^^,^^4^^,^^5^^,^^6^ The right choice of the DSB repair mechanism is crucial for the correct restoration of the DNA molecule. HR requires a well-aligned sister chromatid to be error-free, thus it is prioritized when DSBs occur in the S and G_2_ phases of the cell cycle. By contrast, NHEJ is favored in G_1_ phase, when HR would tend to use error-prone templates such as homologous chromosomes. Cells regulate the choice between NHEJ and HR primarily on the basis of cyclin-dependent kinase (CDK) activity, since this correlates well with the absence or presence of a sister chromatid. NHEJ is preferred in G_1_, exactly when CDK activity is low, whereas HR is ubiquitously upregulated by CDK as its activity rises from S phase.^7^^,^^8^ However, as cells transit through M phase, the relationship between CDK, HR, and DSB repair becomes more obscure, despite CDK activity remaining relatively high until the telophase-G1 transition.^9^^,^^10^^,^^11^^,^^12^^,^^13^ Most complex eukaryotes, including animal and plant cells, undergo a mitotic cell division in which chromosomes condense to a large extent in early M phase (prophase-metaphase), concomitant with the resolution of the sister arms. The last chromosomal region to resolve is the centromere at the onset of anaphase, when chromosome segregation occurs. This massive condensation and early arm resolution appears to be incompatible with HR, which is largely inhibited.^6^^,^^9^^,^^11^^,^^12^^,^^13^^,^^14^^,^^15^ In contrast, in simple eukaryotes such as yeast and other fungi, chromosomes barely condense in early M phase (referred to here as G2/M) and are resolved as they segregate in anaphase by an unzipping centromere-to-telomere mechanism.^12^ As a result, sister chromatids remain aligned and suitable for HR until the anaphase onset.^16^^,^^17^

DSB signaling and repair has been studied primarily in G1, S, G2, and early M (G2/M in yeast). Nevertheless, how cells respond to DSBs occurring in the window that spans from anaphase onset to the telophase-G1 transition is poorly known. In higher eukaryotes, this lack of knowledge stems from technical limitations to synchronize cells after the anaphase onset. However, this is not an inconvenience in the yeast *Saccharomyces cerevisiae*, in which cells can be stably arrested in late anaphase and telophase by means of conditional mutants for the mitotic exit network (MEN). The kinase Cdc15 is critical for MEN, and *cdc15* mutants arrest cells in a late anaphase/telophase stage with most sister chromatids apparently resolved and segregated.^18^^,^^19^ Hereafter, we will refer to this *cdc15* stage as late mitosis (late-M). We have previously used this arrest to question how cells respond to DSBs in a state of high CDK but segregated sister chromatids, and found that sister chromatids partly reverse their segregation, allowing for *de novo* sister chromatid alignments that may serve for error-free HR repair.^20^ Accordingly, mutants for HR were as hypersensitive to DSBs in late-M as in G2/M.

In the present work, we sought to identify specific determinants of the DSB response in late-M that may differ from those previously reported in G2/M. To this end, we used comparative abundance proteomics and identified a small set of proteins whose levels are increased upon DSBs in late-M relative to G2/M. Among these, we found the poorly characterized meiotic sister chromatid 1 (Msc1) protein, originally reported to be important for channeling meiotic HR toward the homologous chromosome rather than the sister chromatid.^21^ We confirmed proteomics results by both Western blotting and microscopy, and genetically demonstrate that Msc1 particularly enhances the DSB repair in late-M. Importantly, we show that Msc1 negatively regulates hypersignaling of DSBs and positively regulates the formation of Rad52 factories, which establishes a novel regulatory player of DSB repair in late-M.

## Results

### Abundance proteomics identifies Msc1 as a protein significantly enriched after DSBs in late mitosis

**Figure 1:**
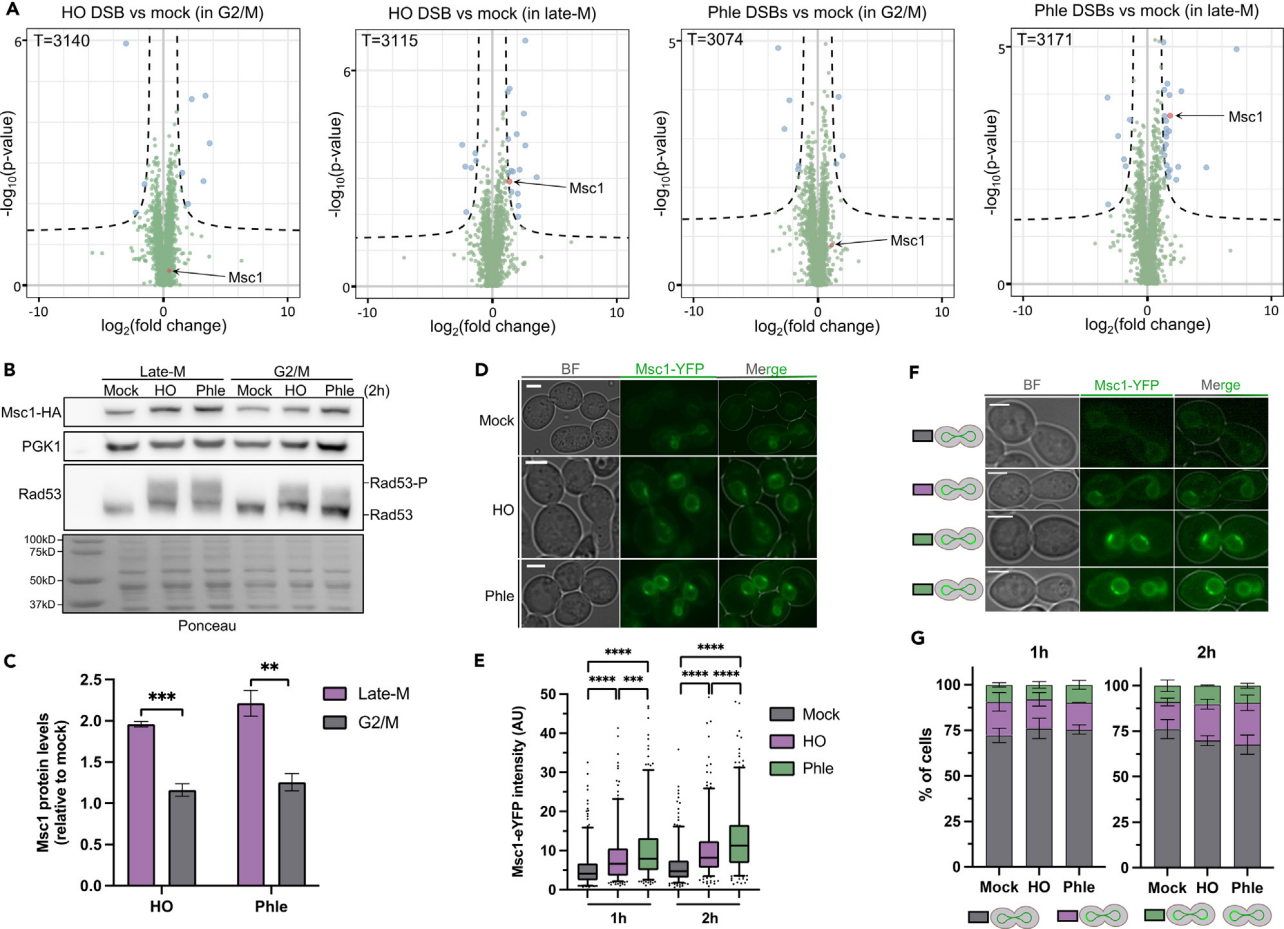
Proteomics for DSBs and Msc1 levels and distribution in G2/M and late mitosis (A) Volcano plots showing the fold change after DNA damage. For each cell cycle phase, the proteomic results of each type of DSB generated (HO and phleomycin) are compared separately with the mock control. Blue dots, enriched or depleted proteins; green dots, background proteins; red dot, Msc1. Total (T) detected proteins is indicated for each plot as well. (B) Western blot to confirm the Msc1 proteomics results. Ponceau staining of the membrane after transfer is also included. (C) Quantification of Msc1-HA after generating DSBs (relative to mock; mean ± SEM, *n* = 3). The statistical analysis was performed by unpaired t test. (D) Representative micrographs of Msc1-eYFP in the mock experiment, HO- and phleomycin-mediated DSBs; 2 h after DSB induction. BF, bright field. Scale bars correspond to 3 μm. (E) Quantification of Msc1-eYFP nuclear envelope levels in late mitosis, 1 and 2 h after DSB generation (mock, HO, and phleomycin). The boxplots correspond to the pull of three independent experiments; >100 cells were quantified in each condition and experiment. Mann-Whitney tests were used for statistical significance. (F) Representative micrographs of NE patches of Msc1. These examples are taken from the mock experiment, and include from top to bottom, cells with no patches, with just one patch, and two patches (either in a single nuclear half or between both halves). Scale bars correspond to 3 μm; BF, bright field. (G) Quantification of Msc1 NE patches and their distribution in the late mitotic nucleus (mean ± SEM, *n* = 3). Gray bars, no patches; purple bars, one patch; green bars, multiple patches. See also Figures S1–S3 and Tables S1 and S2.

To screen for proteins that may be specifically involved in DSB signaling and repair at late stages of the mitotic cycle, we designed an experimental setup whereby we compared the proteomes of cells blocked in G2/M and late-M and subjected to DSBs (Figure S1). To further strengthen the DSB specificity of proteome changes, we used two distinct sources of DSBs. On the one hand, we treated cells with phleomycin, a radiomimetic drug that generates multiple randomly located DSBs.^22^ Phleomycin intercalates into DNA and locally generates reactive oxygen species (ROS) that attack and chemically modify the DNA until it breaks apart. On the other hand, we have introduced a genetic modification in the tested strain that allows the inducible expression of the HO endonuclease, which generates one DSB at the *MAT* locus. In late-M, the number of DSBs is two because both sister chromatids are expected to be cut by HO. The expression of HO was driven by the newly developed β-estradiol promoter,^23^ a tight promoter that bypasses the need to change the growth media for expression. In each cell cycle stage, we paired the DSB treatment with the corresponding mock treatment (Figure 1A). In this way, we filtered out changes in protein levels due to DSB-independent factors, such as those needed for the cell cycle arrests.

The comparative proteomes revealed a number of proteins that were significantly enriched after DSB generation in both G2/M and late-M (Figure 1A; Table S1; Data S1). Late-M resulted in more significant changes than G2/M, and the effect of phleomycin was stronger than that of HO induction. It is important to note that phleomycin is expected to modify the proteome in three ways: (i) as a DSB generator itself, (ii) as a ROS producer, and (iii) as a xenobiotic. The products of five genes were specifically enriched in late-M after DSB generation with both phleomycin and HO; namely, *TFS1* (*YLR178C*), *GPH1* (*YPR160W*), *GND2* (*YGR256W*), *GDB1* (*YPR184W*), and *MSC1* (*YML128C*). Of these five, Msc1 is the only one that has been previously implicated in DSB signaling and repair, specifically in the choice between sister chromatids and homologous chromosomes during meiotic HR.^21^ None of the well-established factors involved in DSB signaling and repair were significantly enriched in any condition (Table S2; Data S1). This suggests that constitutive levels of DSB proteins are sufficient to cope with DSBs, and this interpretation correlates well with previous transcriptomic data in which mRNA levels of these proteins changed little after DNA damage.^24^ Alternatively, any increase could be masked by post-translational modifications of DSB proteins that would affect their detection by mass spectrometry (e.g., phosphorylation, ubiquitination, sumoylation, etc.). Accordingly, our proteomics analysis detected about half of the proteins encoded in the yeast genome (∼3,000 proteins), but DSB repair proteins appeared clearly underrepresented (Table S2; Data S1). Since Msc1 had been linked to HR before, we decided to focus on this particular protein for the rest of this work.

### Msc1 is nuclear envelope protein whose levels increase in late mitosis after DSBs

We began by validating the proteomic data that pointed toward a 4-fold increase of Msc1 in late-M after DSBs (2-fold increase after log2 transformation). To do this, we tagged Msc1 at the C-terminus with the HA epitope and measured Msc1 levels by western blotting (Figures 1B and 1C). In these western blots, we also included a housekeeping control for normalization, Pgk1, as well as the DSB sensor Rad53 as a reporter since it becomes hyperphosphorylated after DSB generation.^25^ Msc1 was enriched 2-fold after either phleomycin or HO treatments when compared to mock treatments. This enrichment was late-M specific as Msc1 barely changed after DSBs in the G2/M arrest.

Next, we investigated whether the increase of Msc1 levels was post-translationally regulated or as a result of an increase in *MSC1* expression. Hence, we measured *MSC1* mRNA levels by RT-qPCR in all tested conditions (Figure S2A). We found that mRNA levels increased slightly after DSB generation (∼1.5 in late-M and ∼1.25 in G2/M). Although neither the mRNA increase was as high as that of the protein levels nor the cell cycle specificity was as remarkable, the general mRNA trend was in good agreement with that of the Msc1-HA western blots, suggesting that the Msc1 increase after DSBs is mostly regulated at the transcriptional level. Consistent with this, we could not detect major post-translational modifications after DSBs (Figure S2B).

In addition to western blots, we decided to address Msc1 levels by microscopy, which can report on the protein location and any shift that may occur after DSB generation (Figures 1D, 1E, and S3). We tagged Msc1 with either eYFP or mCherry and found it at the nuclear periphery (nuclear envelope and/or perinuclear endoplasmic reticulum) in asynchronous and synchronized populations (Figures 1D and S3). This NE location was further confirmed in a strain triple-labeled with Msc1-eYFP, the nucleoporin Nup49-mCherry (as NE marker) and the histone Hta2-CFP (as chromatin marker) (Figure S3A). Interestingly, Msc1-eYFP abundance at the single cell level appeared to be highly variable, spanning up to 30-fold in NE intensity (Figure 1E, mocks), with cells where Msc1 was barely visible and cells with a very intense NE signal (Figures 1D and S3B–S3D). Both phleomycin and HO treatments led to a steady increase in Msc1-eYFP abundance (Figures 1D and 1E), until reaching, for example, a 4-fold change after 2 h in phleomycin. In addition, in approximately 25% of the cells, Msc1 appeared unevenly distributed along the nuclear periphery, presenting concentrated patches (Figures 1F and 1G). The number of patches varied between one (∼15%) and two (∼10%); when two were present, they could be in either the same or opposite nuclear bodies in the elongated late-M nucleus. However, we did not observe any change in the proportion of cells with non-uniform Msc1 distribution after generating DSBs (Figure 1G).

### Cells lacking Msc1 are hypersensitive to DSBs

**Figure 2:**
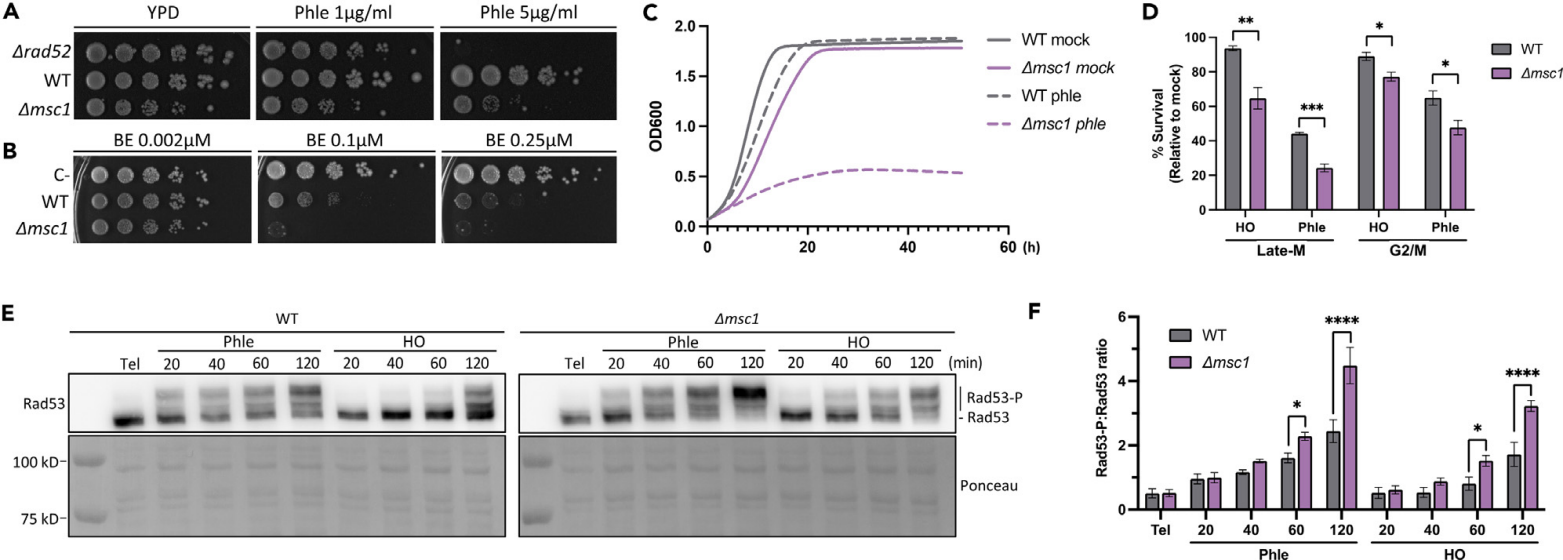
Sensitivity of *Δmsc1* to DNA damage (A) Spot assay on phleomycin. A rad52Δ strain served as a positive control for sensitivity to DSBs. (B) Spot assay for HO DSBs. Both WT and Δmsc1 strains carry the HO endonuclease under the control of the β-estradiol (BE) promoter. The rad52Δ strain used in (A) served here as a negative control (C-) as it does not carry the BE-HO system. (C) Growth curves of WT and Δmsc1 with and without phleomycin (*n* = 2). The SEM is not shown for clarity (its highest value was less than 0.1). (D) Late-M versus G2/M clonogenic survival of WT and Δmsc1 after DSBs (mean ± SEM, *n* = 3). Survival was estimated relative to mock treatments (unpaired t test). (E) Western blots of Rad53 hyperphosphorylation (Rad53-P) after DSBs in the WT and the Δmsc1 strains. Tel, telophase (i.e., late mitosis). (F) Quantification of the Rad53-P:Rad53 ratio over the time course (mean ± SEM, *n* = 3; unpaired t test). See also Figures S5 and S6.

Having validated that Msc1 levels increase after DSBs in late mitosis, we next addressed whether cells deficient in this protein are more sensitive to DSBs. We first attempted to conditionally degrade Msc1 using the auxin-based aid degron system (Figure S4). In this manner, and even though Msc1 is not essential for cell proliferation,^21^ we could restrict the absence of Msc1 function to the short window of late-M DSBs. Unfortunately, Msc1-aid was not degraded in the presence of the auxin, hence we used the Δ*msc1* knockout mutant instead. Sensitivity tests based on spot assay experiments showed that the Δ*msc1* strain was more sensitive to phleomycin than its isogenic wild-type (WT) counterpart (Figure 2A). Similarly, Δ*msc1* was also hypersensitive to the DSB generated by the HO endonuclease (Figure 2B). For phleomycin, a similar hypersensitive profile was obtained in the growth curves (Figure 2C). Lastly, the Δ*msc1* strain was also hypersensitive to other forms of DNA insults that do not initially generate DSBs, although they do in the long term such as the replication stress drugs hydroxyurea (HU) and methyl methanesulfonate (MMS) (Figure S5A). In contrast, the mutant did not cf. hypersensitivity to oxidative stress (Figure S5B). The latter profile reinforces that the phleomycin hypersensitivity is directly due to DSB formation and not to the concomitant production of ROS.

Although spot assays and growth curves clearly demonstrated the hypersensitivity of Δ*msc1* to DSBs, they could not discriminate whether this sensitivity was cell cycle specific, particularly whether or not late-M Δ*msc1* cells were more sensitive than their G2/M counterparts. To address this, we proceeded as shown in Figure S1 and determined the percentage of cells surviving DSBs in late-M and G2/M through clonogenic assays (Figure 2D). Relative to mock, the decrease in viability in the WT after HO induction was only 10% in both G2/M and late-M, whereas it was 40% and 60% in phleomycin, respectively. In the case of Δ*msc1*, these decreases were higher, and more severe in late-M than in G2/M (2-fold less viability in Δ*msc1* than in the WT in late-M, versus just 1.3-fold in G2/M).

### DNA damage signaling is higher in Δ*msc1*

To elucidate the molecular basis of the DSB hypersensitivity in Δ*msc1*, we first determined the kinetics of the DNA damage checkpoint (DDC). Rad53 is a master kinase in the DDC that is found hypophosphorylated in cells that are not experiencing DNA damage.^25^ By contrast, in the presence of ongoing DNA damage, including DSBs, Rad53 becomes hyperphosphorylated, and this molecular change is easily detected by Western blotting as an electrophoretic shift and the appearance of multiple slow-migrating Rad53 bands. Cells blocked in late-M prior to DSB generation showed a hypophosphorylated band (Figure 2E),^20^ and once cells were either exposed to either phleomycin or HO expression, Rad53 became hyperphosphorylated. When we compared the kinetics of Rad53 phosphorylation in the WT and the Δ*msc1*, we observed that Rad53 became more hyperphosphorylated in the latter (Figures 2E and 2F), especially at the later time points of the experiment (1 h and 2 h).

To check whether this increase was due to a deficiency in DDC shutdown in Δ*msc1*, we repeated the DSB generation by HO induction, but washed out β-estradiol after 1 h, thus allowing late-M cells to recover from the DSB insult. HO is known to be rather unstable and is rapidly degraded once the HO promoter is silenced.^26^^,^^27^^,^^28^ Rad53 remained hyperphosphorylated for the first 3 h after recovery and was gradually dephosphorylated over the next 6 h (Figure S6). By 18 h after recovery, Rad53 had reached the hypophosphorylated state seen before DSBs (or in the parallel mock treatments). Relative to the WT, no apparent delay in dephosphorylation kinetics was observed in Δ*msc1*.

### DSB repair by homologous recombination is slower in Δ*msc1*

**Figure 3:**
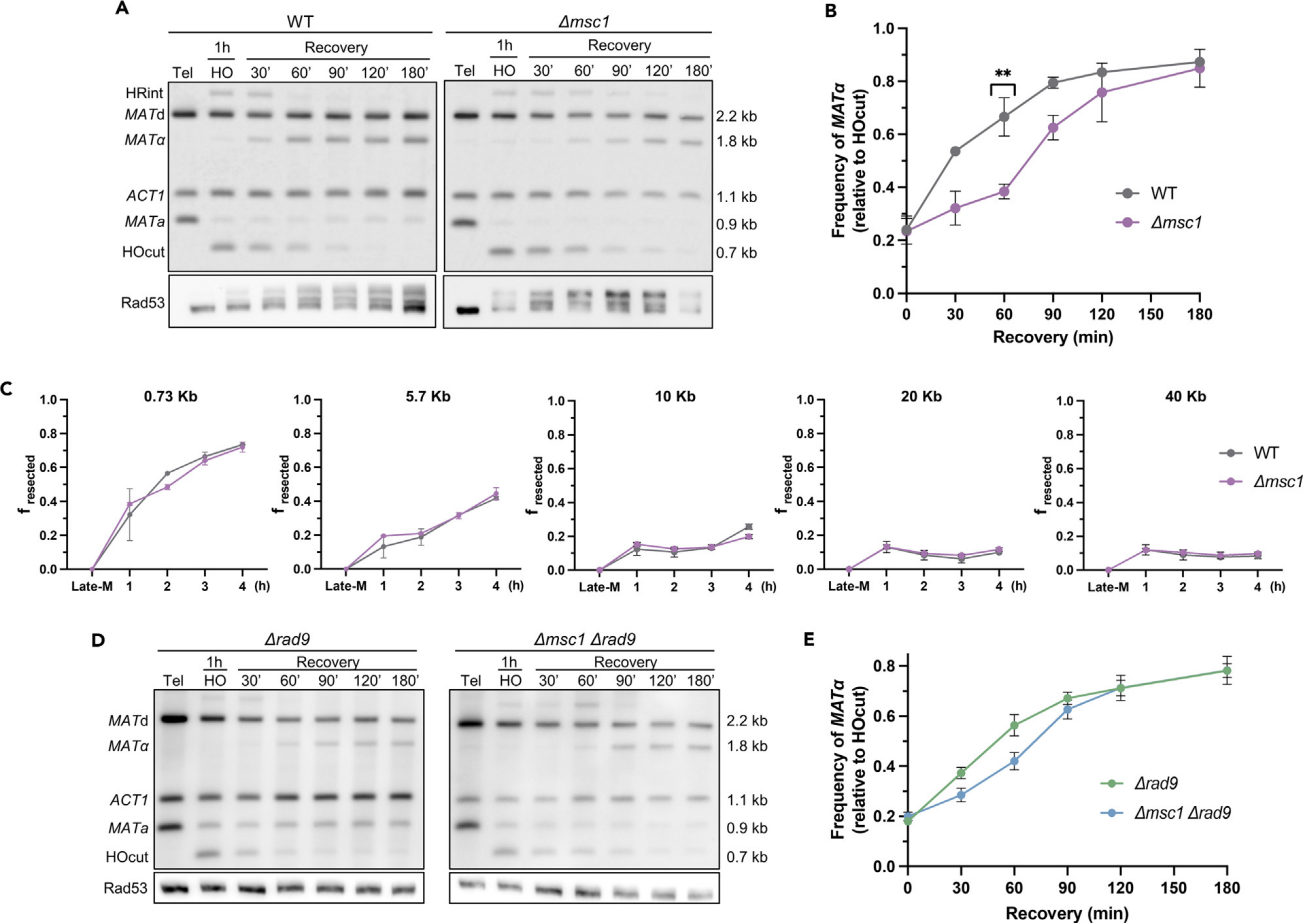
Late mitotic repair of the HO-mediated DSB in the WT and the *Δmsc1* strains (A) Representative Southern blots of the MAT switch assay in the WT and Δmsc1 strains in late-M. The Rad53 western blot of the experiment is shown below. Tel, telophase (i.e., late mitosis). (B) Quantification of the MATa conversion into MATα in the WT and Δmsc1 strains (mean ± SEM, *n* = 3). The switch was normalized to the amount of the MATa cut after HO induction (the HOcut band). The unpaired t test at 60′ is shown. (C) Resection kinetics for amplicons located downstream of the HO-generated DSB (mean ± SEM, *n* = 2); f_resected_ is the fraction of resected DNA. (D) Representative Southern blots of the MAT switch assay in the Δrad9 and Δrad9 Δmsc1 strains in late-M. Note how Rad53 is not phosphorylated in the western blot shown below. Tel, telophase (i.e., late mitosis). (E) Quantification of the MATa conversion into MATα in the Δrad9 and Δrad9 Δmsc1 strains (mean ± SEM, *n* = 3). The switch was normalized as in (B). See also Figures S7–S10.

The next issue we addressed was the kinetics of the DSB repair. To do this, we made use of the MAT switching system, a well-established reporter that allows both quantification of the DSB repair process and how much of it goes through either HR or NHEJ (Figure S7A).^29^ In G2/M, the HO DSB is efficiently repaired through HR.^16^^,^^17^ Importantly, the HO DSB is also repaired by HR in late mitosis.^28^ To determine whether Msc1 impinges on the overall repair of the HO DSB in late-M, we compared the WT and the Δ*msc1* strains (Figures 3A and 3B). In both strains, most cells have efficiently cut the *MAT*a sequence after just 1 h of HO expression. Upon removal of the HO, the DSB began to be repaired by HR, yielding the *MAT*α product in more than 80% of the cases by 2 h. There was no difference between the WT and the Δ*msc1* mutant at this time; however, during the first 1.5 h, there was a clear delay in the Δ*msc1* (Figures 3B and S8). This points out that Msc1 ensures an early repair of DSBs by HR, which may be critical for fast-cycling cells such as these, especially during the rapid transition through mitosis. No signs of repair through NHEJ were observed; however, to fine tune whether any DSB could be channeled toward NHEJ, we measured the *MAT*a band throughout the experiment in derivative strains in which the *RAD52* gene had been deleted (Figure S9). Rad52 is an essential HR player,^30^ and gene conversion to *MAT*α is fully dependent on Rad52.^31^ NHEJ was absent in late-M in both *MSC1* Δ*rad52* and Δ*msc1* Δ*rad52* (Figure S9).

In order to channel DSBs for HR repair, DSB ends must first be resected, this is, processed into single-stranded DNA (ssDNA) so that these ssDNA tracts can search for homology in other genomic regions.^32^ To test whether resection was affected in Δ*msc1*, we compared resection efficiency along one of the HO DSB ends (Figure 3C). We measured resection based on a qPCR strategy capable of detecting ssDNA as it becomes resistant to *StyI* digestion (Figure S7B). For this experiment, we maintained HO expression throughout, observing that resection frequencies were mostly equivalent in both the WT and the Δ*msc1* mutant (Figure 3C). As expected, the closer to the DSB the faster the resection was. Only at 10 kb from the DSB and after 4 h, a small increase was observed in the WT relative to the Δ*msc1* mutant. In *rad52*Δ, resection appears to be even more efficient (Figure S10), probably because of the inhibitory role of Rad52 on part of the resection machinery.^33^ Importantly, however, there was no difference between *MSC1* Δ*rad52* and Δ*msc1* Δ*rad52*.

Rad53 hyperactivation has been linked to negative feedback on the resection kinetics.^34^ Thus, it might occur that the slower DSB repair in the Δ*msc1* mutant stems from such hyperactivation. To look into this, we deleted the checkpoint adaptor gene *RAD9*. In the Δ*rad9* mutant, Rad53 remains hypophosphorylated and inactive even after generating DSBs (Figure 3D). Without the checkpoint, HR repaired the HO DSB, although the overall kinetics were slightly slower in *MSC1* Δ*rad9*. Importantly, there was still a delay in Δ*msc1* Δ*rad9* relative to *MSC1* Δ*rad9*, and the overall kinetics of the latter was not affected (Figure 3E). This points out that Msc1 ought to facilitate HR by acting on the HR process itself, and that the checkpoint hyperactivation must be a consequence of the delay, rather than the cause.

### Msc1-deficient cells contain fewer Rad52 repair factories after DSBs

**Figure 4:**
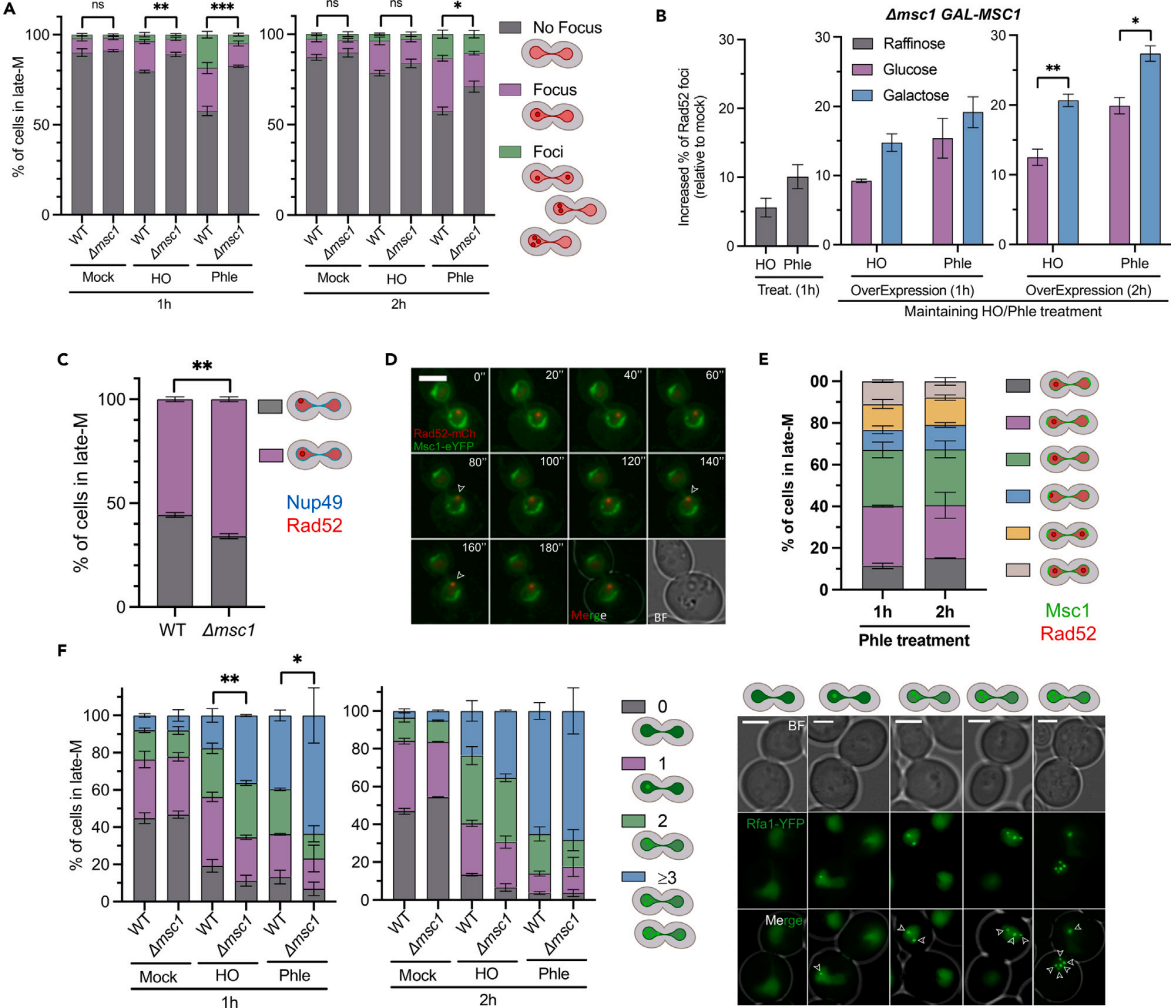
Msc1 affects the number and location of Rad52 repair factories in late mitosis (A) Quantification of the absence of presence of late-M Rad52 foci after DSBs (mean ± SEM, *n* = 3; unpaired t test on “no focus” category). (B) Effect of Msc1 overexpression on Rad52 foci after DSBs (mean ± SEM, *n* = 3; unpaired t test). On the left (gray bars), the increase of late-M cells with Rad52 foci 1 h after DSB generation (relative to mock treatments); in the middle, the Rad52 foci increase 1 h after MSC1 overexpression (and 2 h from DSBs); on the right, the Rad52 foci increase 2 h after MSC1 overexpression (and 3 h from DSBs). The control subcultures without overexpression (glucose) are also shown. (C) Role of Msc1 in the nuclear positioning of Rad52 foci. Strains bearing RAD52:mCherry and NUP49:eCFP were treated as in Figure 1A. After 2h of DSB generation with phleomycin, WT and Δmsc1 late-M cells bearing Rad52-mCherry and Nup49-eCFP were categorized based on whether (gray bars) or not (purple bars) a single Rad52 focus was in contact with the NE reporter Nup49 (mean ± SEM, *n* = 3). (D) Dynamics of Rad52 location in late-M cells with DSBs. A WT late-M cell with a single Rad52-mCherry focus at the periphery (as determined by Msc1-eYFP) was filmed for 3 min (20″ frames). Note how the Rad52 dynamically detaches from the NE (arrowheads indicate transitional foci-NE contact). This example corresponds to a 2 h treatment with phleomycin. (E) Distribution of Rad52 foci relative to Msc1 patches in late-M cells with phleomycin-driven DSBs (mean ± SEM, *n* = 3). The quantified categories include: (i) cells with a single Rad52 focus and Msc1 patches localized to either one or both segregating nuclear bodies (dark gray, purple, green, and blue bars); and (ii) cells with two Rad52 foci in different nuclear bodies (yellow and light gray bars). The category of a single Rad52 focus and a single Msc1 patch in the same nuclear body was further subdivided into those cells where foci and patches are in contact (blue bar) and those where they are not (green bar). (F) Quantification of late-M Rfa1 foci before and after DSBs (mean ± SEM, *n* = 3; unpaired t test on “>3 foci”). In all panels, statistical significance was calculated by an unpaired t test (n.s. indicates no significant differences; ∗ indicates *p* < 0.05; ∗∗ indicates *p* < 0.01; and ∗∗∗ indicates *p* < 0.001). In micrographs, scale bars correspond to 3 μm; BF, bright field. See also Figures S11–S13 and Table S3.

Having shown that DSB resection is not altered in Δ*msc1*, yet HR products are only obtained at later time points, we turned our attention to HR events that occur downstream. Resected DSBs are eventually coated by HR proteins involved in the search for homologous sequences and the formation of HR intermediates.^5^ All of these processes occur at distinct sites in the nucleus, which are referred to as DNA repair factories and where these HR factors are concentrated. These factories can be visualized by tagging HR proteins with fluorescent proteins.^35^ The most widely used representative of these factories is Rad52; thus, we followed Rad52-mCherry in late-M before and after DSBs (Figures 4A and S11A). We found that around 10% of cells arrested in the *cdc15-2* late-M presented Rad52 foci prior to DSB generation, and these values did not change during the mock treatments (Figure 4A). The percentages were equal for the WT and the Δ*msc1* strain. In the WT, HO-generated DSBs increased this percentage to ∼25%, whereas in phleomycin this percentage was even higher (∼45%). Interestingly, the amount of Δ*msc1* cells with Rad52 foci was significantly lower in both DSB scenarios, especially 1 h after DSB generation (∼15% and ∼20%, respectively). After 2 h, the fractions of Δ*msc1* cells with foci approached the WT values. We thus concluded that Msc1 facilitates the recruitment of Rad52 to DSBs.

We also examined Rad52 foci in the Δ*rad9* derivatives (Figure S12). We found that there was a significant increase in the number of cells with foci after phleomycin addition, and that the differences between *MSC1* and Δ*msc1* were dampened and not significant. This suggests that overresection might rescue the ability to recruit Rad52 to DSBs in Δ*msc1*, as unabated resection is one of the main consequences of depleting Rad9.^36^

Seeking to strengthen a functional relationship between Msc1 and Rad52, we next tested whether Msc1 overexpression could increase the number of Rad52 foci after DSBs in the Δ*msc1* background. For this purpose, we constructed strains where *MSC1* was under the control of the *GAL1-10* promoter (Figure S13A). This is a strong and inducible promoter which can be rapidly switched on by shifting the carbon source of the growth medium from raffinose to galactose. In WT strains, Msc1 levels were higher than endogenously expressed Msc1 as early as 1 h after galactose addition (Figure S13B). These levels were even much higher after 2 h in galactose. Long-term Msc1 overexpression was toxic in both spot assays and growth curves (Figures S13C–S13E), and overexpressed Msc1 did not rescue WT sensitivity to phleomycin in growth curves (Figure S13D). However, in short-term experiments with Δ*msc1* cells arrested in late-M, overexpression of Msc1 for 2 h increased the proportion of cells with Rad52 foci in both HO and phleomycin (Figures 4B and S11B).

### Msc1 NE patches and Rad52 foci tend to colocalize in the same nuclear body

Functional HR repair factories are normally located well within the nucleus and away from the nuclear periphery.^37^ Factories may also be targeted to the NE when the DSBs are unrepairable or otherwise destined to be repaired by BIR.^38^^,^^39^^,^^40^ On the basis of the location of Msc1 at the nuclear periphery, we compared the spatial distribution of Rad52 foci between the WT and the Δ*msc1* strains. To do this, we added the NE reporter Nup49, and nuclear pore complexes (NPC) protein, to our Rad52-mCherry. In the *cdc15-2* arrest, around 40% of all Rad52 foci localized to the periphery, in close proximity to the Nup49-eCFP NE signal (Figures 4C and 4D). This proportion is slightly higher than expected for a random distribution,^39^^,^^40^ yet probably is a consequence of the higher surface-to-volume ratio expected in the extended nucleus of late mitosis. In Δ*msc1*, the percentage of peripheral Rad52 decreased to 30%. Although the difference was not as pronounced, it was significant. Time-lapse movies of Rad52 foci in the late mitotic arrest showed that the NE location (as seen with Msc1-eYFP) is dynamic, with factories rapidly moving in and out of the NE (Figure 4D). Hence, two non-mutually exclusive explanations may account for the 10% decrease in Δ*msc1*; fewer HR factories are assembling at the periphery but not in the interior and/or moving factories spend less time associated with the NE. In either case, it is clear that the slower DSB repair in Δ*msc1* is not a consequence of a relocalization of Rad52 factories toward the NE.

As mentioned previously, Msc1 location across the NE in late mitosis is asymmetric, with cells exhibiting patches of enriched Msc1. Thus, we next examined whether there was an association between Rad52 foci and Msc1 patches. Foci were more frequently found in arrested cells with patches (Table S3). In the subset of cells with both foci and patches, the proportion of cells with a single patch and a single focus colocalizing in the same nuclear body was far greater than the 1:1 ratio expected if they were independent events (Figure 4E; 3.5:1 ratio; the sum of green and blue versus dark gray bars). In spite of this, Rad52 foci were rarely in visual contact with the Msc1 patch (Figure 4E; blue bar).

### There are temporarily more RPA foci in Msc1-deficient cells after DSBs

Once resection exposes the 3′ ssDNA, this is rapidly protected by coating it with the RPA complex. Later on, this complex is replaced by Rad51 through the action of Rad52.^32^ Since, we observed similar resection but fewer Rad52 foci in Δ*msc1*, we investigated the kinetics of RPA after DSBs. Similar to Rad52, RPA forms nuclear foci after DSBs.^35^ Thus, we tagged Rfa1, one of the three subunits of RPA in *S. cerevisiae*, with YFP, and repeated the experiment shown above for Rad52-mCherry. In general, there were more cells with Rfa1-YFP foci and more foci per cell (Figure 4F). This is, however, consistent with previous results from others.^35^ In the WT, foci were already present in the late-M mock, as observed before.^41^ The Δ*msc1* strain showed a similar RPA pattern in the mock treatment. For the WT and Δ*msc1*, the number of cells with foci and the number of foci significantly increased after both HO induction and phleomycin addition. Remarkably, this predominantly occurs in the Δ*msc1* strain 1 h after DSB generation, with differences disappearing after 2 h (Figure 4F). This result points out that Msc1 may aid in the transition between the resected ssDNA coated by RPA and the assembly of Rad52, presumably for the formation of the Rad51 filament.

## Discussion

Repair of highly deleterious DSBs by HR requires an intact sister chromatid for not being mutagenic. Prior to DNA replication, when a sister is not yet available, HR is inhibited as its activity is coupled to CDK activity.^7^^,^^8^ However, in the final stages of mitosis, after sister chromatid segregation, CDK activity still remains high and thus HR remains active. In a previous work, we observed that *S. cerevisiae* resolves this paradox by approaching and coalescing the segregated sister, which could give HR one last chance to repair faithfully. We have now used a proteomic approach to search for new factors that can play a prominent role in DSB repair in late-M. From this study, we have identified the loosely characterized protein Msc1 (Figure 1A; Table S1). After validating the proteomic findings by western blotting and microscopy (Figures 1B–1E), we confirmed that the deletion mutant is more sensitive to DSBs generated in late-M (Figures 2A–2D).

**Figure 5:**
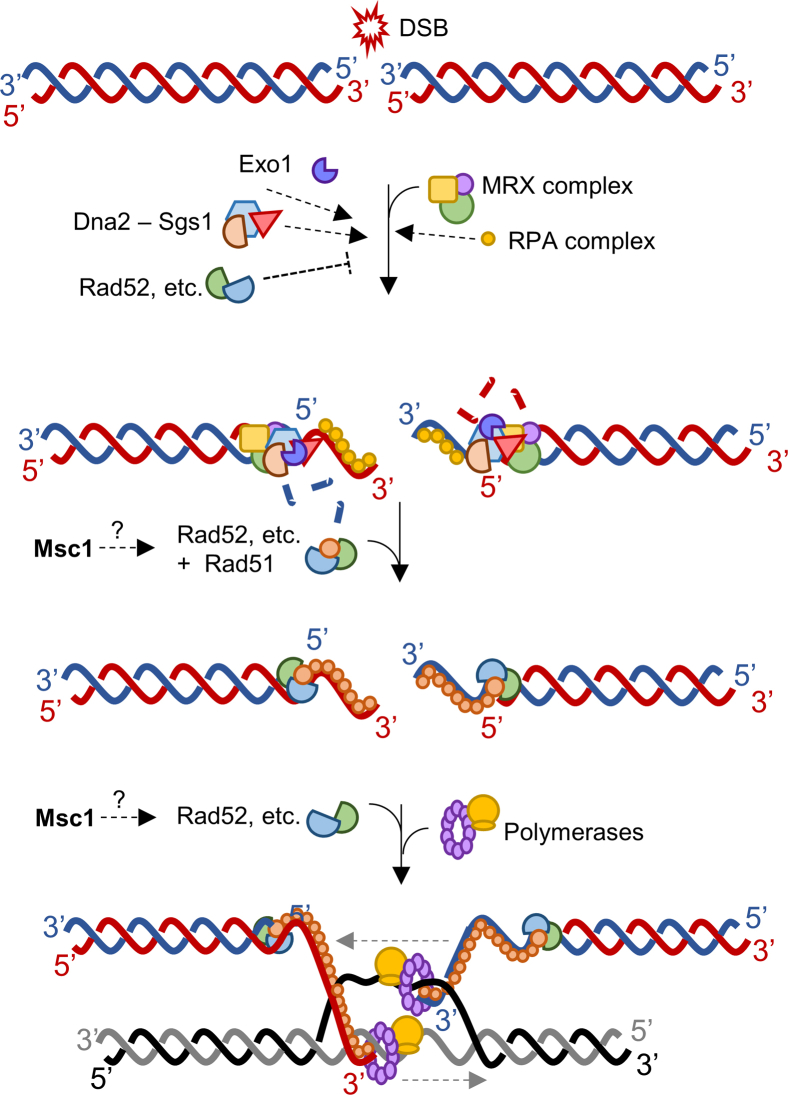
Model of the putative position of Msc1 in the HR pathway The 5′ ends of DSBs are recognized and resected by MRX, Exo1 and Dna2-Sgs1 and then coated by RPA. Rad52 modulates the resection, the replacement of RPA by Rad51 and the invasion of the template donor. Msc1 enhances a step between resection and invasion.

HR is a complex stepwise process that initiates with DNA end resection, where nucleases degrade the 5′ ends, generating 3′ ssDNA tails, which are protected from degradation by the RPA complex. Rad51, aided by Rad52 (BRCA2 in humans) and other accessory proteins, forms a synaptic filament on the ssDNA, which requires displacement of the RPA complex (Figure 5).^32^ The Rad51-ssDNA filament then searches and invades a homologous duplex DNA, forming a displacement loop (D loop). The invasion primes DNA synthesis using the intact homologous sequence as a template, leading to branch migration. After DNA synthesis, the recombination intermediates can be resolved in different ways. From a mechanistic point of view, the Δ*msc1* mutant appears to affect either the formation of the presynaptic filament or later stages (Figure 5). Accordingly, HR completion is delayed compared to WT (Figures 3B and 3C), but end resection kinetics is not (Figure 3D). It does occur a minor delay in initiating resection (Figure S8A), but most of the delay befalls once resection has started and ensuing recombination intermediates are seen (Figure S8B). This position downstream of the resection also fits well with the fact that the mutant has fewer Rad52 repair centers (Figure 4A), but at the same time has higher signs of DNA damage (hyperphosphorylated Rad53) (Figures 2E, 2F, and S7). In addition, late-M cells accumulate more RPA foci after DSBs in Δ*msc1* mutant (Figure 4F), which is consistent with delaying the formation of the ssDNA-Rad51 filament. Similarly, in the Δ*rad9* mutant, where resection is unrestrained,^34^^,^^36^ a similar delay is observed in the Δ*rad9* Δ*msc1* (Figures 3D and 3E). Nevertheless, as HR is not a lineal irreversible process, and also Rad52 participates in events downstream of the ssDNA-Rad51 formation, we cannot rule out that Msc1 may act during the search and invasion of homologous template or the new DNA synthesis that extends the D loop (Figure 5). It is worth noting that the search for the template is probably the most challenging step in late-M cells. In favor of this scenario, we observed that DSB end over-resection attenuated the differences in Rad52 foci formation between *MSC1* and Δ*msc1*, but did not change the overall kinetics of the repair (Figures 3D, 3E, and S12).

Msc1 is an NE protein that belongs to the poorly characterized Ish1 family. To date, the best characterized member of this family is Les1 from *Schizossacharomyces pombe*, which has been linked to aberrant karyokinesis.^42^ SpLes1 is a nuclear envelope protein that localizes to the bridge stalk before karyokinesis and corrals NPC in late-M.^42^ Because SpLes1 and ScMsc1 are orthologs, the Msc1 deficiency could also interfere with the axis that regulates the repair of DSBs via NPCs. In *S. cerevisiae*, this axis is particularly important for DSBs that lack a nearby template^40^^,^^43^^,^^44^^,^^45^, as is the case in late-M. In this sense, DSB targeting to the NE and the putative role of Msc1 in NPC dynamics may facilitate the search for distant templates, perhaps overcoming the barrier of the long and thin nuclear bridge.^20^ Alternatively, the lack of Msc1, and the corresponding loss of an orderly distribution of NPCs in late-M, could affect the biochemical control that NPCs have over HR proteins; e.g., through sumoylation.^46^ Further research will shed light on these mechanistic insights.

In conclusion, through abundance proteomics we have identified a novel protein, Msc1, that enhances DSB repair in late mitosis, when the nucleus is elongated and sister chromatids have been segregated. Our data further suggest that Msc1, which is an NE protein, upregulates the assembly of Rad52 repair factories and accelerates HR completion. This work presents for the first time to the best of our knowledge a protein with a specific role in late-M HR.

### Limitations of the study

Despite the identification of Msc1 as a protein required for late-M DSB repair and its encasement in the linear pathway of HR, a molecular mechanism connecting Msc1 with HR remains elusive. Moreover, this study was conducted in a model eukaryote that undergoes a close mitosis, in which the NE is not disassembled in late-M. Given that Msc1 is itself an NE protein, the results of this study may be limited to organisms with a close mitosis and may not be directly applicable to human cells.

## STAR★Methods

### Key resources table

**Table:** 

REAGENT or RESOURCE	SOURCE	IDENTIFIER
**Antibodies**		

Primary mouse monoclonal anti-HA (1:1000)	Sigma-Aldrich	H9658; RRID:AB_260092
Primary mouse monoclonal anti-Myc (1:5000)	Sigma-Aldrich	M4439; RRID:AB_439694
Primary mouse monoclonal anti-Pgk1 (1:5000)	Thermo Fisher Scientific	22C5D8
Primary mouse monoclonal anti-miniaid (1:500)	MBL	M214-3; RRID:AB_2890014
Primary mouse monoclonal anti-Rad53 (1:1000)	Abcam	ab166859; RRID:AB_2801547
Polyclonal goat anti-mouse conjugated to horseradish peroxidase (1:5000; 1:10000; or 1:20000)	Promega	W4021; RRID:AB_430834
Anti-fluorescein antibody coupled to alkaline phosphatase	Roche	11426338910; RRID:AB_514504

**Bacterial and virus strains**		

Escherichia coli: DH5α	Invitrogen	18265017

**Chemicals, peptides, and recombinant proteins**		

Phleomycin	Sigma-Aldrich	P9564
β-estradiol	Sigma-Aldrich	E8875
Nocodazole	Sigma-Aldrich	M1404
3-indol-acetic acid	Sigma-Aldrich	I2886
PAGE Laemmli Sample Buffer	Bio-Rad	1610747
β-mercaptoethanol	Sigma-Aldrich	M3148
PVFD membranes	Pall Corporation	PVM020C099
Ponceau S solution	PanReac AppliChem	A2935
ECL reagent	GE Healthcare	RPN2232
PowerUp™ SYBR™ Green Master Mix	Thermo Scientific	A25741
Sty-I-HF	NEB	R3500S
Lyticase	Sigma-Aldrich	L4025
Phenol:chloroform:isoamylalcohol	PanReac AppliChem	A0944
RNase A	Roche	10109169001
Positively charged nylon membrane (Hybond-N+)	Amersham-GE	RPN303B
Fluorescein-12-dUTP Solution	ThermoFisher	R0101
CDP-Star	Amersham	RPN3682
Phos-Tag™ Acrylamide AAL-107	FUJIFILM Wako	300-93523

**Critical commercial assays**		

MoClo Yeast Toolkit	Lee et al.^47^	AddGene Kit#1000000061
PureLink™ RNA Mini Kit	Thermo Scientific	12183018A
High Capacity RNA-to-cDNA kit	Thermo Scientific	4387406
Expand™ High Fidelity PCR System	Roche	11732641001
PureYield™ Plasmid Miniprep System (250 preps)	Promega	A1222
Qubit™ Protein Assay Kit	Thermo-Scientific	Q33212

**Deposited data**		

*S. cerevisiae* protein database	ENSEMBL	version R64-1-1.24
Mass spectrometry proteomics data. ProteomeXchange Consortium via PRIDE. (https://www.ebi.ac.uk/pride/archive)	This paper	PXD043515
Unprocessed blots and microscopy fields, results for each biological replicate. Mendeley data (https://data.mendeley.com/)	This paper	https://doi.org/10.17632/gbwywn92gg.1

**Experimental models: Organisms/strains**		

*Saccharomyces cerevisiae*. Strains background: YPH499.	See Table S4	See Table S4

**Oligonucleotides**		

Primer ACT1 forward: TGTCACCAACTGGGACGATA	This paper	ACT1-F(+533)
Primer ACT1 reverse: AACCAGCGTAAATTGGAACG	This paper	ACT1-R(+813)
Primer MSC1 forward: TTGGATGACATAAAGGGTTG	This paper	MSC1-C-tag_CHK-F
Primer MSC1 reverse: CACCGAATGATTTTAGGTAC	This paper	Msc1-R_int
Primer re section 20kb from HOcs forward: CCGATGAACTTCAGAATCCTCG	This paper	20kb dsHOcs_F
Primer re section 20kb from HOcs reverse: ATGCATTAGTAATGGCGTTCAGG	This paper	20kb dsHOcs_R
Primer resection 40kb from HOcs forward: GGAAGGCATTTACCCCTACCAAAAC	This paper	40kb dsHOcs_F
Primer resection 40kb from HOcs reverse: GAAGTGCCCGTTTACCTAAAGCAAC	This paper	40kb dsHOcs_R
All the other primers used in resection assay	Gnügge et al.^48^	ADH1, HOcs, 727pb, 5.7kb and 10kb

**Recombinant DNA**		

Plasmid:*[ColE1::KanR::ura3::GAL1p:MSC1:mTURQUOISE2:tADH1::URA3::ura3]*	This paper	N/A

**Software and algorithms**		

MaxQuant Software	Cox and Mann^49^	https://www.maxquant.org/.
R	The R core team	https://www.r-project.org/
BioProfile Bio1D (v15.07)	Vilber-Lourmat	https://www.vilber.com/
ImageJ/Fiji	NIH	https://imagej.net/software/fiji/
Zen Blue edition (v3.2)	Zeiss	https://www.zeiss.com/microscopy/en/home.html
Prism (v9 and v10)	Graphpad	https://www.graphpad.com/features
QuantStudio Desing & Analysis software (v2.5)	ThermoFisher Scientific	https://www.thermofisher.com/es/es/home.html
EvolutionCapt	VilberLourmat	https://www.vilber.com/
Microsystems LAS AF6000	Leica	https://www.leica-microsystems.com/
Spark Control Magellan (v3.0)	TECAN	https://lifesciences.tecan.com/software-magellan
SnapGene Viewer	Dotmatics	https://www.snapgene.com/snapgene-viewer

**Other**		

MSC1 Moclo type 3 gBlocks HiFi Gene fragments	IDT	Sequence available upon request
Qubit 4 Fluorometer	ThermoFisher Scientific	Q33227
48-pin replica plater	Sigma-Aldrich	R2383
Fusion Solo S chamber	Vilber-Lourmat	N/A
QuantStudio5 Real-Time PCR instrument	ThermoFisher Scientific	N/A
Zeiss Axio Observer.Z1/7. Axiocam 702 sCMOS camera, the Colibri-7 LED excitation system. Plan-Apochromat 63x/NA 1.40 Oil M27 DIC objective.	Zeiss	N/A
Spark incubator	TECAN	N/A
Exploris 480 mass spectrometer coupled to an Easy-nLC 1200 system	ThermoFisher Scientific	N/A

### Resource availability

#### Lead contact

Further information and request for resources and reagents should be directed to and will be fulfilled by the lead contact, Félix Machín (fmachin@fciisc.es).

#### Materials availability

All materials generated in this study are available from the lead contact upon request.

#### Data and code availability

• Proteomics data have been deposited at ProteomeXchange Consortium via the PRIDE partner repository and are publicly available as of the date of publication. Accession numbers are listed in the key resources table.
• This paper does not report original code.
• Raw data from main and supplemental figures were deposited on Mendeley at https://doi.org/10.17632/gbwywn92gg.1. Any additional information required to reanalyze the data reported in this paper is available from the lead contact upon request.

### Experimental model and study participant details

#### Yeast strains and experimental conditions

All yeast strains used are listed in Table S4. Strain construction was undertaken through standard transformation methods.^50^ Gene deletions and C-terminal tags were engineered using PCR methods.^51^ The MoClo Yeast Toolkit was used to create the *MSC1* overexpression plasmid following the instructions.^47^ To add *MSC1* to the system as a type 3 module, the sequence of *MSC1* with the necessary overhangs was ordered as a synthetic gene (gBlocks HiFi Gene fragments from IDT). A synonymous mutation was also added to the sequence to destroy a target for the restriction enzyme BsaI, which is used for the assembly of the different modules.

Strains were grown overnight in air orbital incubators at 25°C in YPD media (10 g·L^−1^ yeast extract, 20 g·L^−1^ peptone and 20 g·L^−1^ glucose) unless stated otherwise. To arrest cells in late mitosis, log-phase asynchronous cultures were adjusted to OD_600_ ∼ 0.4 and the temperature was shifted to 34°C for 3 h. In most experiments, the arrested culture was split into three subcultures: one subculture was treated with phleomycin (2-10 μg·mL^−1^; Sigma-Aldrich, P9564), a second one with β-estradiol (2 μM; Sigma-Aldrich, E8875) for the induction of HO endonuclease,^52^ and the third was just treated with the vehicle (mock treatment). In general, samples were collected at the moment of the arrest and at 1 and 2 h after DNA damage. The general experimental setup is depicted in the upper branch of Figure S1. In the case experiments to analyze DNA repair (MAT switching and Rad53 inactivation), all cultures were washed twice with fresh YPD and further incubated for 4-24 h to recover from DNA damage. In resection experiments, β-estradiol was added to the culture at the late mitotic arrest and maintained for 4 h.

To synchronize cells in G2/M, 15 μg·mL^−1^ nocodazole (Nz; Sigma-Aldrich, M1404) was added and the cells held at 25°C for 3 h, with a Nz boost (7.5 μg·mL^−1^) at 2 ht. Then, G2/M cultures were treated as described above for the late mitotic arrest. In experiments with conditional degron variants for the auxin system (aid tags), the protein was targeted for degradation by adding 5 mM 3-indol-acetic acid (IAA; Sigma-Aldrich, I2886) 1 h prior to adding phleomycin or β-estradiol. When galactose induction was required, cells were grown in YP raffinose 2% (w/v) as the carbon source and galactose was added at 2% (w/v) 1 h after the DNA damage induction while keeping the yeast cultures at 34°C.

### Method details

#### Proteomics

The experimental setup shown in Figure S1 was followed for comparative proteomics. After taking the corresponding samples, they were processed for mass spectrometry (MS). Firstly, samples were boiled 10 min at 80°C in LDS buffer with 10 mM DTT. Subsequently, proteins were separated using a pre-casted 4-12% NuPAGE Bis-Tris gel and run at 180 V for 10 min. The samples were later processed by the in-gel digestion protocol described in.^53^ In short, samples were first reduced in reduction buffer (10 mM DTT in 50 mM ammonium bicarbonate buffer (ABC buffer) at 56°C for 1 h and alkylated in alkylation buffer (50 mM iodoacetamide in 50 mM ABC buffer) for 45 min in the dark. Proteins were then digested overnight with 1 μg MS-grade trypsin at 37°C in 50 mM ABC buffer and the digested peptides were eluted onto a C18 StageTip, following the micro-purification protocol from.^54^ Each sample was measured with a 120 min method on an Exploris 480 mass spectrometer coupled to an Easy-nLC 1200 system (ThermoFisher Scientific) with a 50 cm column packed in-house with Reprosil C18. The mass spectrometer was operated with a top20 data-dependent acquisition method.

MS files were processed using the MaxQuant Software and the ENSEMBL *S.cerevisiae* protein database (version R64-1-1.24). The options “LFQ quantification” and “match between runs” were activated. The output files were analyzed using R. First, known contaminants, reverse hits and protein groups only identified by site were removed. Then, identified protein groups were filtered with a minimum of two quantification events per experiment. Missing values were imputed with a downshifted and compressed beta distribution within the 0.001 and 0.015 percentile of the measured values for each individual replicate. For plotting, LFQ intensities were log2 transformed. A two sample Welch t-test was performed. Volcano plots were generated by plotting -log10 (p-values) and fold changes. The threshold line for enriched proteins was defined with p-value=0.05, s=1 and c=0.5.

Finally, we used the fact that in 8 out of 24 samples we had induced the HO endonuclease to internally validate the proteomics readouts. Thus, HO was detected in 7 out of 8 HO induction experiment (HO at G2/M plus HO in late mitosis, n=4 each), whereas it was absent in all the other 16 samples (mock at G2/M, phle at G2/M, mock in late mitosis, and phle in late mitosis; n=4 each) (Data S1).

#### Western blots

Western blotting was carried out as reported before with minor modifications.^20^ Briefly, 5 ml samples were collected, the cell pellets were fixed in 1 mL of 20% (w/v) trichloroacetic acid TCA, and broken by vortexing for 3 min with ∼200 mg of glass beads in 2 mL tubes. Samples were then centrifuged, pellets were resuspended in 150 μL of a mixture of PAGE Laemmli Sample Buffer 1X (Bio-Rad), Tris HCl 0.75M pH 8.0 and β-mercaptoethanol 2.5% (Sigma-Aldrich), and tubes were boiled at 95°C for 3 min and pelleted again. Total protein in the supernatant was quantified using a Qubit 4 Fluorometer (Thermo Fisher Scientific). Proteins were resolved in general in 7.5% SDS-PAGE gels and transferred to PVFD membranes (Pall Corporation). The membrane was stained with Ponceau S solution (PanReac AppliChem) as a loading reference. In the case of Phos-Tag™ (FUJIFILM Wako), the SDS-PAGE gels contained 0.03 mM of this compound and 0.06 mM MnCl. Before the standard transfer procedure, the gel was soaked in transfer buffer with 10 mM EDTA.

The following antibodies were used for immunoblotting: The HA epitope was detected with a primary mouse monoclonal anti-HA (1:1,000; Sigma-Aldrich, H9658); the Myc epitope was detected with a primary mouse monoclonal anti-Myc (1: 5,000; Sigma-Aldrich, M4439); the Pgk1 protein was recognized with a primary mouse monoclonal anti-Pgk1 (1:5,000; Thermo Fisher Scientific, 22C5D8), the aid tag was recognized with a primary mouse monoclonal anti-miniaid (1:500; MBL, M214-3), and Rad53 was recognized with a primary mouse monoclonal anti-Rad53 (1:1000; Abcam, ab166859). A polyclonal goat anti-mouse conjugated to horseradish peroxidase (1:5,000; 1:10,000; or 1:20,000; Promega, W4021) was used as the secondary antibody. Antibodies were diluted in 5% skimmed milk TBST (TBS pH 7.5 plus 0.1% Tween 20). Proteins were detected by using the ECL reagent (GE Healthcare, RPN2232) chemiluminescence method, and visualized in a Vilber-Lourmat Fusion Solo S chamber.

Protein bands were quantified using BioProfile Bio1D software (Vilber-Lourmat) and then normalized with respect to PGK1, which was considered as the housekeeping. Subsequently, the Msc1 level detected in each type of damage was normalized with respect to its mock.

#### qPCR

qPCR was performed on genomic DNA (for resection experiments) and from cDNA (for expression experiments) in 96-well 0.2ml block plates using a QuantStudio5 Real-Time PCR instrument. Reactions had a final volume of 10 μl and were prepared with PowerUp™ SYBR™ Green Master Mix (Thermo Scientific, A25741). The High Capacity RNA-to-cDNA kit (Thermo Scientific, 4387406) was used for the retrotranscription. RNA was extracted using the PureLink™ RNA Mini Kit (Thermo Scientific, 12183018A) and gDNA was extracted using glass beads/phenol Winston’s method.^55^ Each sample for resection analysis was divided into two aliquots and one aliquot was digested with the Sty-I-HF (NEB, R3500S) restriction enzyme. Primers used in the resection assay are listed in.^48^ Primers used in the expression experiments are: 5’-TGTCACCAACTGGGACGATA-3’and 5’-AACCAGCGTAAATTGGAACG-3’ for *ACT1* as control; 5’-TTGGATGACATAAAGGGTTG-3’ and 5’-GTACCTAAAATCATTCGGTG-3’ for *MSC1*.

To calculate *f*_*resected*_, it was first necessary to calculate the fraction of the extracted genomic DNA where HO had cut the MAT locus, which is simply known as *f*. For this, the following equation was used f=1-((E_HOcs_^ΔCq(t0-t)^)/(E_ADH1_^ΔCq(t0-t)^)).^48^ Then, *f*_*resected*_, the fraction in which the resection has passed the restriction site, was calculated with this second equation f_resected_=2/(((E_RS_^ΔCq(digest-mock)^)/(E_ADH1_^ΔCq(digest-mock)^)+1)·f). In both cases, E is the primer efficiency and ΔC_q_ represents the difference in quantification cycles.

#### Microscopy

A Zeiss Axio Observer.Z1/7 was also used. This inverted microscope was equipped with an Axiocam 702 sCMOS camera, the Colibri-7 LED excitation system, narrow-band filter cubes for covisualization of CFP, YFP/GFP, and mCherry without emission crosstalk, and a Plan-Apochromat 63x/NA 1.40 Oil M27 DIC objective.

For each field, a stack of 10-20 z-focal plane images (0.2-0.3 μm depth) was collected. In general, the images were taken from freshly harvested cells without further processing and at least 100 cells were quantified per experimental data point. The Zen Blue (Zeiss) and Fiji-ImageJ (NIH) software were used for image processing and quantification. Scale bars represent 4 μm in all cases.

For Rad52-mCherry and Rad51-YFP factories three distributions were quantified: "No focus" (cells with a homogeneously diffused nuclear Rad52); "Focus" (a single Rad52 spot); and "Foci" (more than one Rad52 spot). For Rfa1-YFP the “foci” category was further split in two, “2 foci” and “>2 foci”.

#### Growth curves and viability analyses

For clonogenic survival assays, log-phase asynchronous cultures were adjusted to OD_600_ = 0.4 before the corresponding block and ensuing treatment. After that, 100 μL of 4:10,000 dilutions were spread onto YPD plates. Viability was measured by plotting the number of colonies grown on the plates after 3 days at 25°C. The mock treatments yielded 400–600 CFU/plate in these experiments.

For spot sensitivity assays cultures were grown exponentially and adjusted to an OD_600_ = 0.5 and then 5-fold serially diluted in YPD in 96-well plates. A 48-pin replica plater (Sigma-Aldrich, R2383) was used to spot ∼3 μL onto the corresponding plates, which were incubated at 25°C for 3–4 days before taking photographs.

For growth curves, strains were first grown exponentially in YPD and then an inoculum was taken and adjusted to an initial OD_600_ = 0.05 in either fresh YPD or YPGalactose (2% w/v), without or with phleomycin (2 μg·mL^−1^). Three replicates of each culture were aliquoted in a flat-bottomed 96-well plate and real-time growth was measured in a Spark TECAN incubator by reading the OD_600_ every 15 minutes for 50 hours with shaking (96 rpm and 6mm of orbital amplitude). The mean of the three replicates was calculated to obtain the final growth curves. Two independent experiments were performed but only one is shown since the SEM was less than 0.1 OD_600_ for each time point.

#### MAT switching assay and southern blots

After taking the samples, genomic DNA was extracted by a lytic method. Briefly, the pellets were resuspended in 200 μl of digestion buffer (1 % SDS, 100 mM NaCl, 50 mM Tris-HCl, 10 mM EDTA and 50U Lyticase (Sigma-Aldrich, L4025)) and incubated at 37°C. DNA was isolated by phenol:chloroform:isoamylalcohol (PanReac AppliChem, A0944), precipitated with ice-cold ethanol 100%, resuspended in TE 1X containing 10 μg.mL-1 RNase A (Roche, 10109169001) allowing the enzyme to act for a short incubation, precipitated a second time and resuspended in TE 1X. Then, the purified DNA was digested with StyI, the restriction fragments were separated on a 1.2% low EEOO LS Agarose gel, and finally Southern blotted. Southern blot was carried out by a saline downwards transference onto a positively charged nylon membrane (Hybond-N+, Amersham-GE; RPN303B) as reported before.^56^ DNA probes against *ACT1* and *MAT*a loci were synthesized using Fluorescein-12-dUTP Solution (ThermoFisher; R0101) and the Expand™ High Fidelity PCR System (Roche; 11732641001). Hybridization with fluorescein-labeled probes was performed overnight at 68°C. The next day, the membrane was incubated with an anti-fluorescein antibody coupled to alkaline phosphatase (Roche; 11426338910), and the signal was developed using CDP-star (Amersham; RPN3682) as the substrate. Detection was recorded using the VilberLourmat Fusion Solo S instrument.

For the quantification of the assays, each individual band was normalized to the *ACT1* signal corresponding to its sample. Then, a second normalization was performed for the signal of each *MAT*α band with respect to the intensity of the HO cut band after one hour of endonuclease action. Consequently, the graphs show the amount of DNA repaired by HR with respect to the total amount of DNA cut by HO.

### Quantification and statistical analysis

Error bars in all graphs represent the standard error of the mean (SEM) of independent biological replicates performed in different days. The number of replicates (n) is given in the figure legend. Graphpad Prism 10 was used for statistical tests. Differences between experimental data points were estimated using the Mann-Whitney U test or the unpaired t-test; the test used is indicated in the figure caption. Significance is denoted by asterisks (∗ indicates p<0.05, ∗∗ indicates p<0.01, ∗∗∗ indicates p<0.001 and ∗∗∗∗ indicates p<0.0001).

In general, we used four types of graphs to represent the data: volcano plots, bar charts, marker line graphs and box plots. In box plots, the center line represents the medians, box limits represent the 25th and 75th percentile, the whiskers extend to the 5th and 95th percentiles, and the dots represent outliers.

## Supplemental information

Document S1. Figures S1–S13 and Tables S1–S4

Data S1. Excel table with proteomics data for volcano plots of Figure 1A, related to Figure 1 and Tables S1 and S2
