# Supplementary material for: Msc1 is a nuclear envelope protein that reinforces DNA repair in late mitosis
Source: iScience. 2024 Jun 11;27(7):110250. doi: 10.1016/j.isci.2024.110250 (PMC11253511; doi:10.1016/j.isci.2024.110250)
Supplement: Document S1. Figures S1–S13 and Tables S1–S4 [file mmc1.pdf]

iScience, Volume *27*

Supplemental information

Msc1 is a nuclear envelope protein

that reinforces DNA repair in late mitosis

Sara Medina-Suárez, Jessel Ayra-Plasencia, Lara Pérez-Martínez, Falk Butter, and Félix Machín

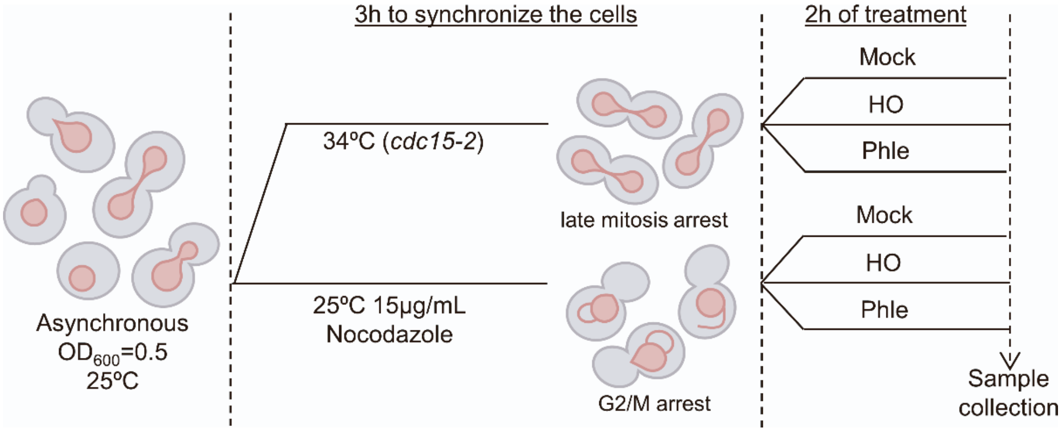

Figure S1, related to Figure 1. Proteomics strategy for DSBs in G2/M and late mitosis. Schematic of the experimental procedure. Cells were first arrested either in G2/M by adding nocodazole or in late mitosis by incubating at 34ºC. Then, the culture was divided into three subcultures. One served as a mock control, whereas the others were treated to generate DSBs, one with β-estradiol (for HO expression) and the other with phleomycin.

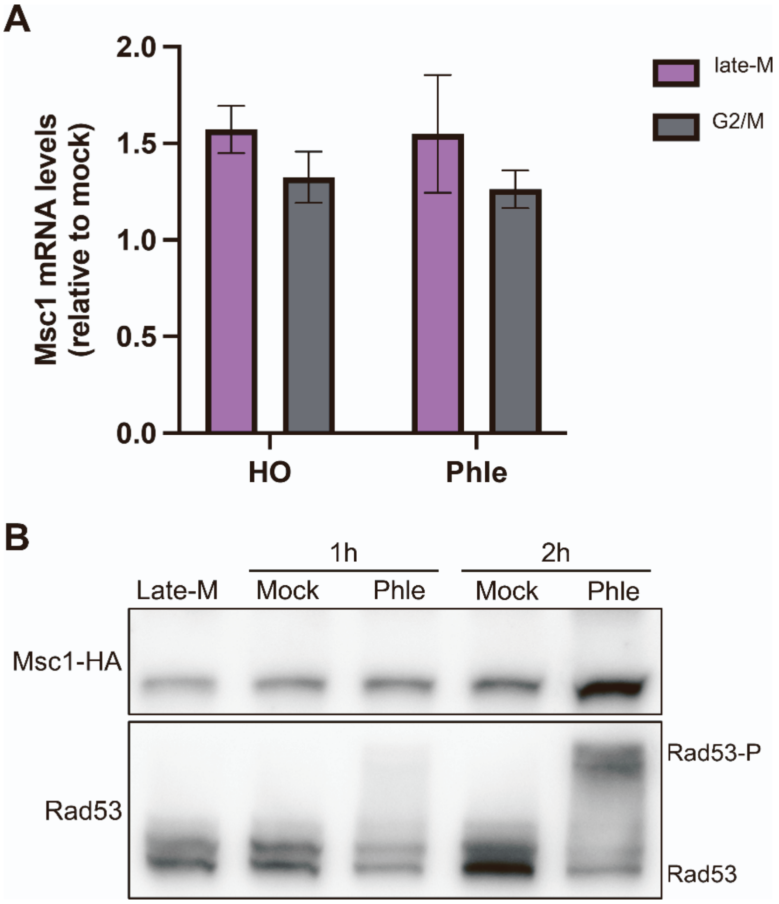

Figure S2, related to Figure 1. *MSC1* mRNA levels and post-translational modifications of Msc1 after DSBs in late-M. (A) Using the same experimental setup as for Western blots in Figure 1C, total RNA samples were collected for RT-qPCR quantification of *MSC1* mRNA levels (mean ± SEM, n=3). (**B**) Post-translational modifications of Msc1-HA after DSBs (phleomycin) were assessed by Western blot in the presence of Phos-Tag™. Note how this condition greatly enhances the shift of hypersphosphorylated Rad53, although no shift was seen for Msc1-HA.

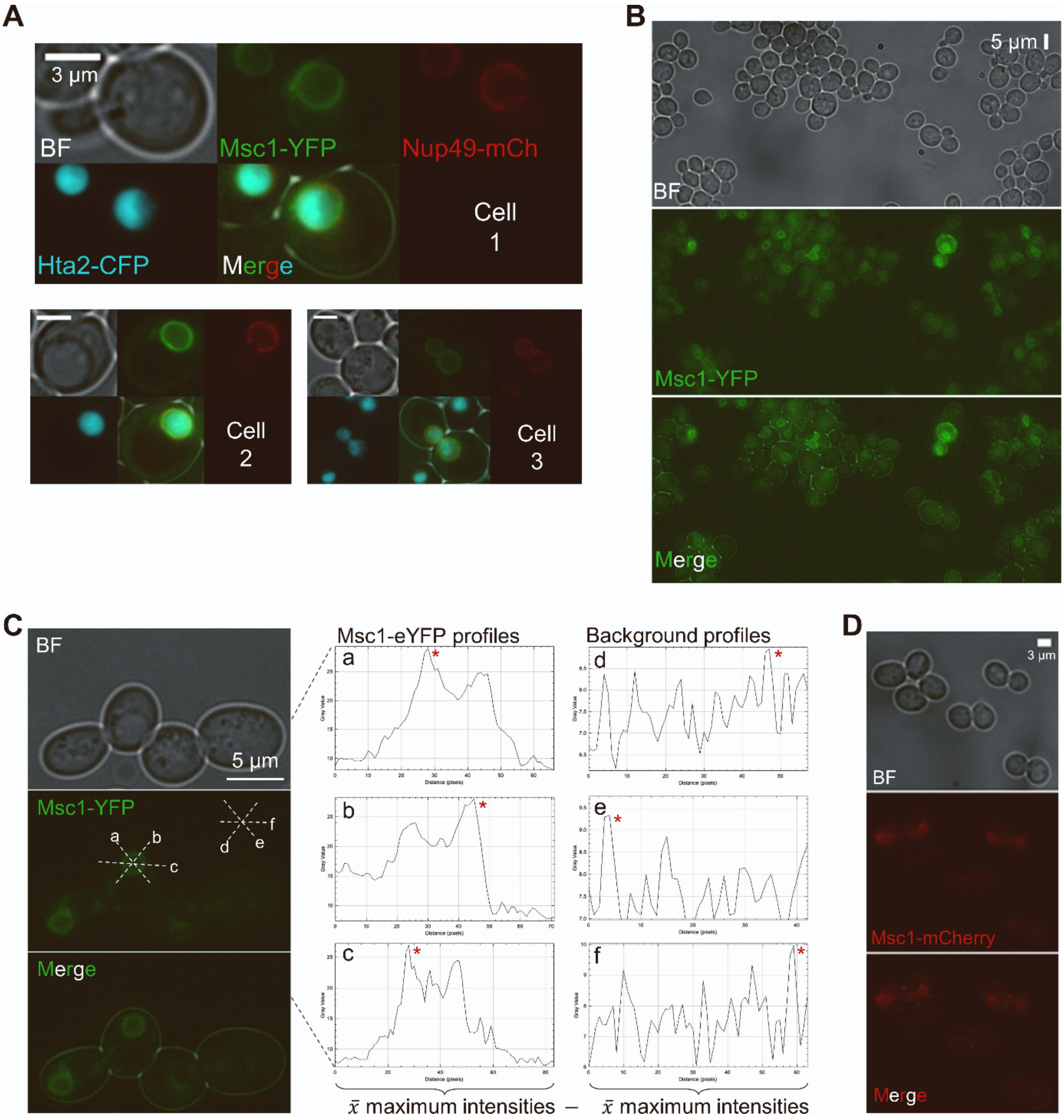

Figure S3, related to Figure 1. Msc1 is a nuclear envelope protein. (A) Representative cells expressing both Msc1-YFP and the NE reporter Nup49-mCherry. All examples taken from an asynchronous population. Note that Msc1 and Nup49 co-localize. **(B)** Representative microscopic field of asynchronous cells expressing Msc1-eYFP under its endogenous promoter. Note that Msc1 localizes to the NE and its levels are variable in the cell population. **(C)** Method for quantifying NE Msc1 levels in late-M cells. Using the ImageJ/FiJi software, each nuclear body in the elongated late-M nucleus was traversed with three selection lines converging at the center and leaving equivalent angles between the lines. Then, the maximum intensity was determined from the intensity profile when the line crossed the NE and the mean of all intensities was calculated. Background intensities were estimated in a similar way in a cell-free area. The NE and background intensities were subtracted to calculate the

relative intensity of each cell. Plots of these intensities are shown in Figure 1E. **(D)** Representative microscopic field of late-M cells expressing Msc1-mCherry.

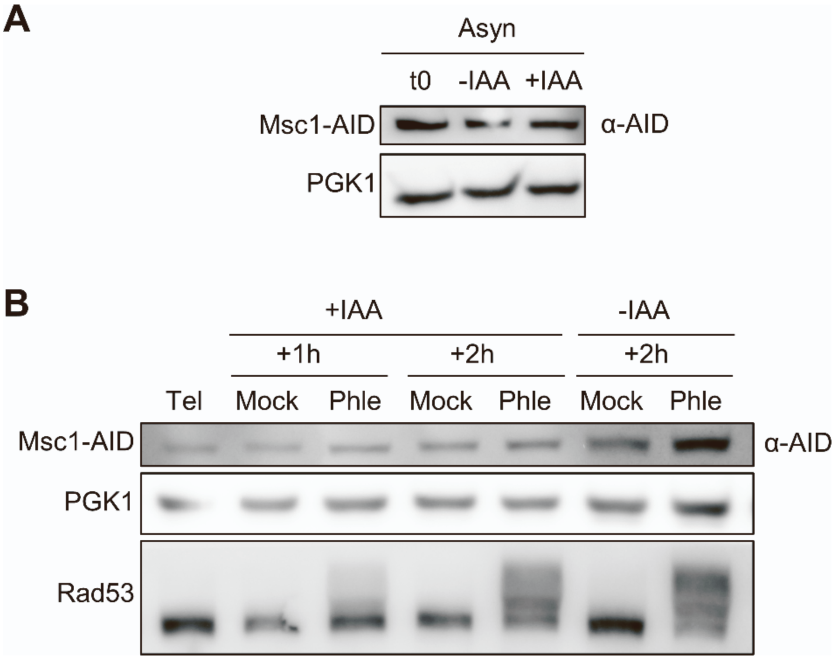

Figure S4, related to Figure 2. Msc1 cannot be degraded using the auxin degron system (AID*). (A) A strain carrying the Msc1-AID* tag and the OsTIR1 gene under the ADH1 promoter was grown overnight in YPD to log phase and then split in two subcultures, which were further incubated for 2 h before taking samples for Western blotting; another sample was taken before the split (t0). 5 mM of the auxin IAA in DMSO 1% v/v was added to one of the subcultures (+IAA) and just DMSO 1% v/v was added to the other one (-IAA). Western blotting was undertaken against either the AID* epitope (α-AID) or the internal PGK1 control. **(B)** A similar experiment was repeated but with cells previously arrested in late-M (34 ºC for 3h). After the arrest, the culture was divided into two subcultures, and 5mM IAA was added to one of them. Each of these subcultures was again split into two; one was then treated with phleomycin to see whether the response to DNA damage modulates the auxin-driven Msc1-aid. From all subcultures, samples were collected at the indicated times. In addition to the anti-AID and the anti-PGK1 antibodies, the anti-Rad53 antibody was included to confirm DNA damage in the phleomycin-treated subcultures. Note that Msc1-aid could not be degraded by IAA under any of these conditions.

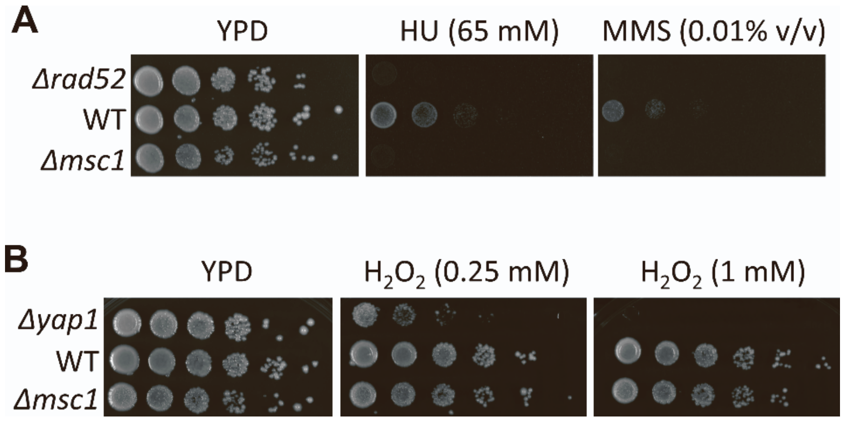

Figure S5, related to Figure 2. Sensitivity of the *msc1*Δ mutant to replicative and oxidative stresses. (A) Spot assays against the replicative stress agents hydroxyurea (65mM) and MMS (0.01% v/v). The first line corresponds to a *rad52*Δ mutant used as a positive control for sensitivity to DNA damage. **(B)** Spot assays against the oxidative stress agent hydrogen peroxide (0.25 and 1mM). The first line corresponds to a *yap1*Δ mutant used as a positive control for sensitivity to oxidative stress. In both cases, the different strains were grown overnight to log phase in YPD, the OD600 was adjusted to 0.5, 1:5 serial dilutions were made and then spotted onto the corresponding plates.

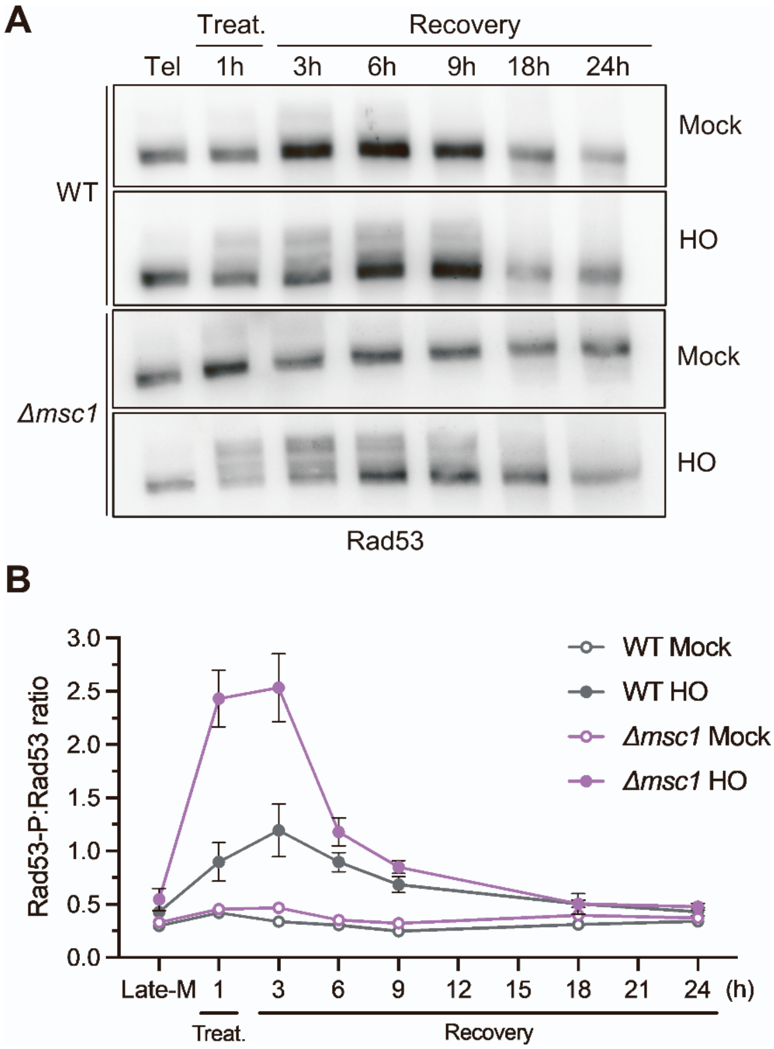

Figure S6, related to Figure 2. Role of Msc1 in DNA damage recovery. (A) Western blot of the loss of Rad53 hyperphosphorylation after DSB recovery in the WT and the *msc1*Δ strains. The experimental setup was as is Figure S1, but only late-M HO-mediated DSBs were generated with 2 µM BE. After 1h, BE was washed off and samples were collected after 3, 6, 9, 18 and 24 h. **(B)** Quantification of the Rad53-P:Rad53 ratio through the time course (mean ± SEM, n=3).

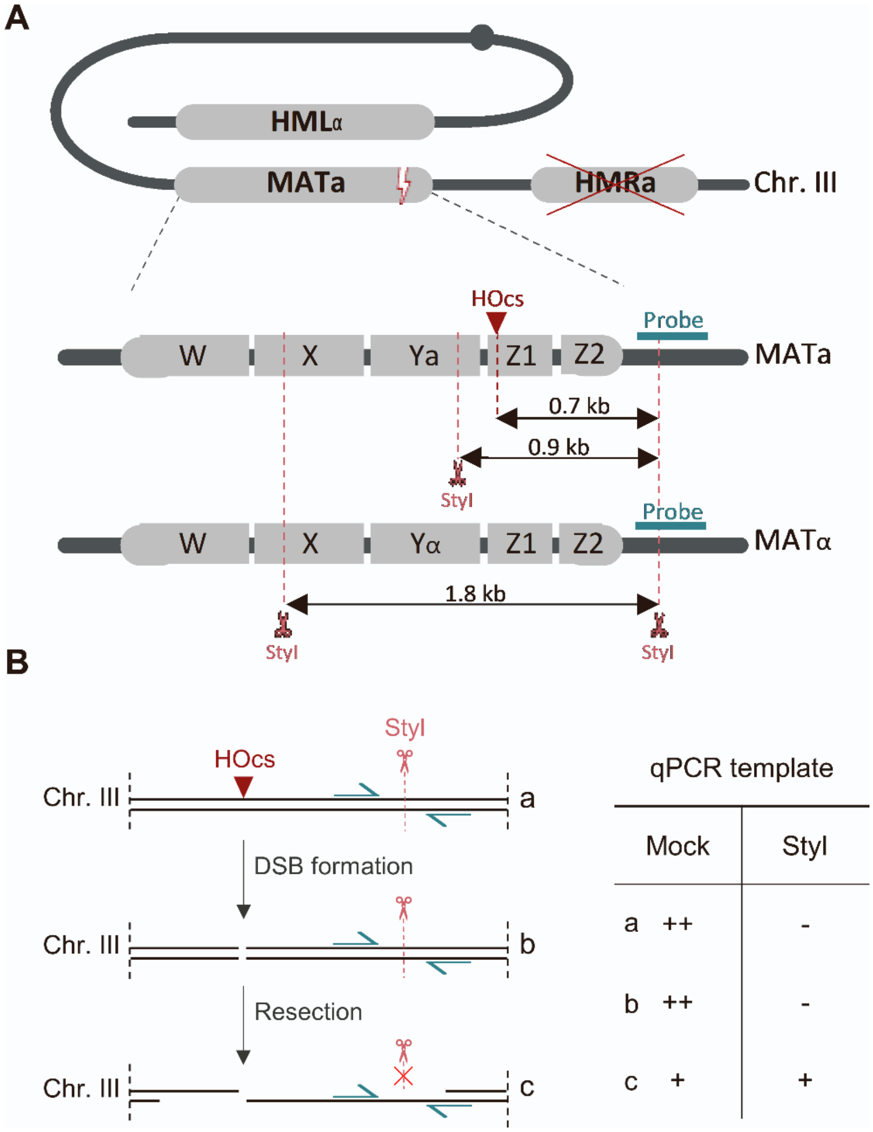

Figure S7, related to Figure 3. The principles of the MAT switching and HO resection assays. (A) Schematic of the *MAT* switching system used in this work. On the top, the bent arrangement of chromosome III for intramolecular HR between the *MAT*a and the *HML* loci is shown. The position of the DSB in the *MAT*a locus is indicated by a lightning sign; red crossed crosses indicate that the alternative recombinogenic *HMR* locus is deleted. At the bottom, a zoomed view of the *MAT* alleles is depicted with the approximate location of the *StyI* restriction sites. Cutting with StyI differentiates the *MAT*a and *MAT*α alleles by Southern blot (fragment sizes are indicated by lines with double arrowheads; the probe is shown in blue). The pattern of *StyI* recognition sequences along the alleles, together with the position of the probe, allows the identification of the uncut *MAT*a (0.9 Kb band), its HO-cut downstream product (0.7 Kb band), and the HR-driven gene conversion to *MAT*α (1.8 Kb band). The probe partially overlaps the most downstream *StyI* site and thus recognizes other sequences downstream of the *MAT* locus. This results in another high molecular weight band (the slowest to migrate; 2.2 Kb) that is affected by neither the actual *MAT* allele nor the HO cut and can be used as an internal loading control for quantification. However, since this

region is relatively close to the HO cutting site (*HOcs*) and could be affected by DSB resection, which could reduce the amount of signal given by the probe, a second probe for a distant locus (*ACT1*, which yields a 1.1 Kb band) is used as a second internal loading control. The cut *MAT*a locus can lead to only three outcomes in the *hmr*Δ strain: (i) gene conversion by HR (the HO-cut 0.7 Kb band disappears in favor of the *MAT*α 1.8 Kb band); (ii) NHEJ (the HO-cut 0.7 Kb band disappears in favor of the *MAT*a 0.9 Kb band); or (iii) the DSB remains unrepaired (neither the 1.8 Kb nor the 0.9 Kb band get enriched relative to their values at the time of the HO removal). **(B)** Diagram of the qPCR assay used to measure resection at the *HOcs*. On the left, schematic of the *HOcs* resection and its effects on PCR amplification. Primers (blue arrows) are designed to amplify sequences containing targets for StyI cleavage. On the right, a summary table of the expected amplification yield (a) before the HO cut, (b) after the HO cut but with resection not reaching the *StyI* site, and (c) with resection extending beyond the *StyI* site. Mock, no StyI digestion; Digested, StyI digestion; ++, extensive amplification, +, moderate amplification, - no amplification.

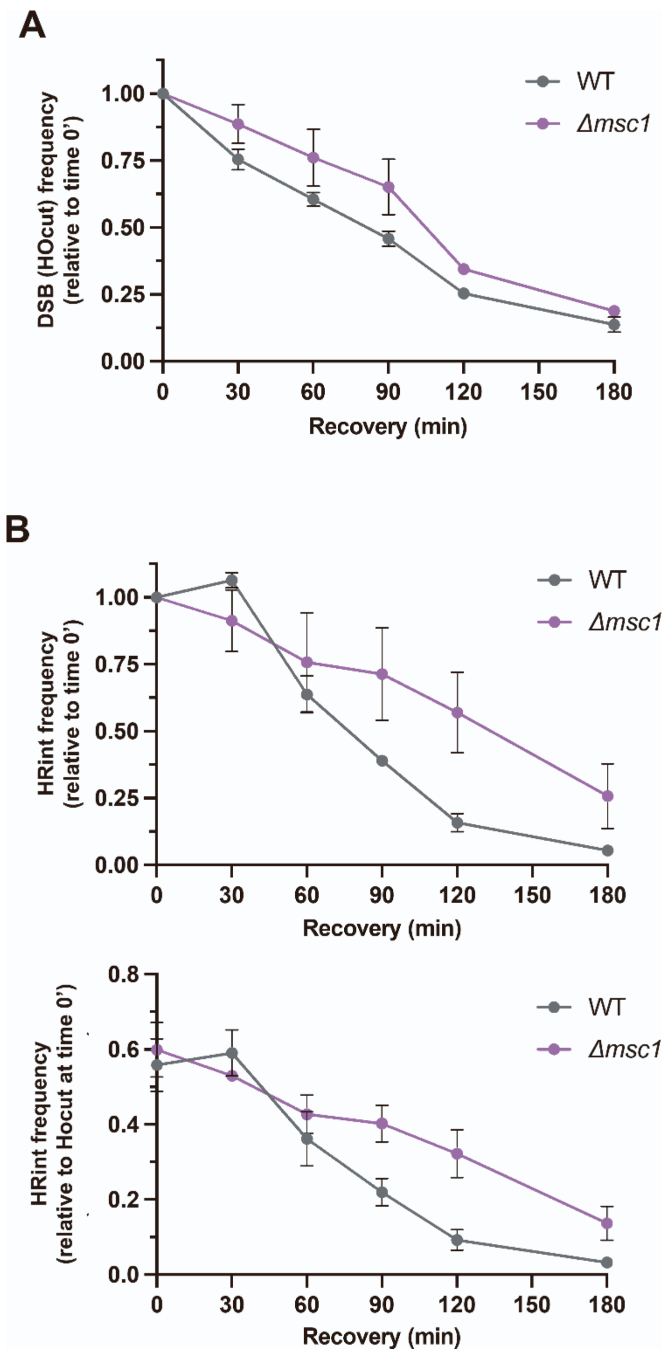

Figure S8, related to Figure 3. Kinetics of the unresected HO DSB and HR intermediates during the DSB repair. (A) Quantification of the HOcut band (unresected DSB) through the experiment (mean ± SEM, n=3). The band was normalized to the amount of HOcut when the HO induction was shut down (time 0'). Note that there is a slight delay in the initiation of the DSB repair in *msc1*Δ. **(B)** Quantification of HR intermediates (slow migrating bands that appear above the MATd band after HO induction; see Figure 3A). HRint throughout the time course was normalized to HRint at time 0'. Note that HR intermediates take longer to resolve into the HR product in *msc1*Δ (see also Figure 3B). **(C)** As in panel B, but normalizing to the HOcut band at time 0'.

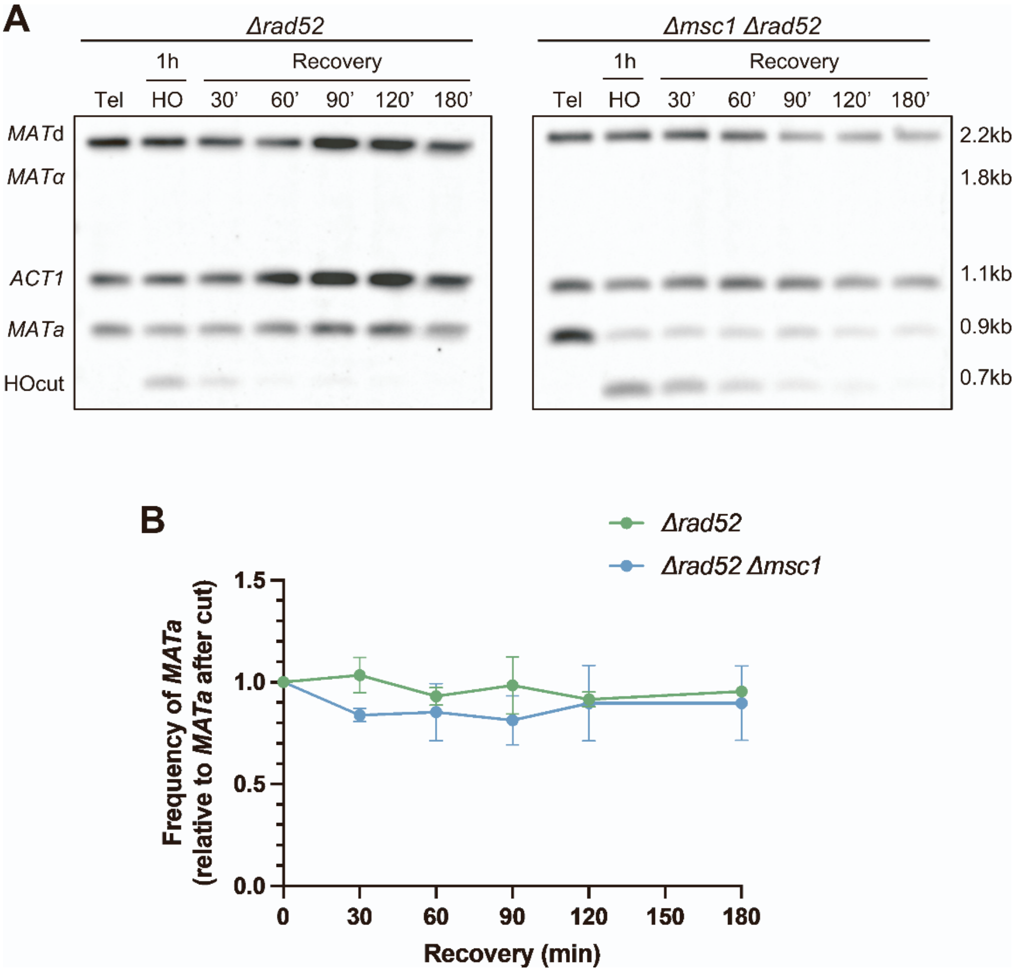

Figure S9, related to Figure 3. Late mitotic repair of the HO-mediated DSB entirely depends on HR. (A) Representative Southern blots of the MAT switching assay in the *MSC1 rad52*Δ and *msc1*Δ *rad52*Δ strains. Note that the *MAT*α HR product is not obtained. (**B**) Quantification of the *MAT*a band through the experiment (mean ± SEM, n=3). The band was normalized to the amount of the *MAT*a that remained after HO induction. Note that even in these *rad52*Δ strains, unable to repair by HR (i.e., no *MAT*α band), no signs of NHEJ are seen either (*MAT*a increase during the DSB recovery).

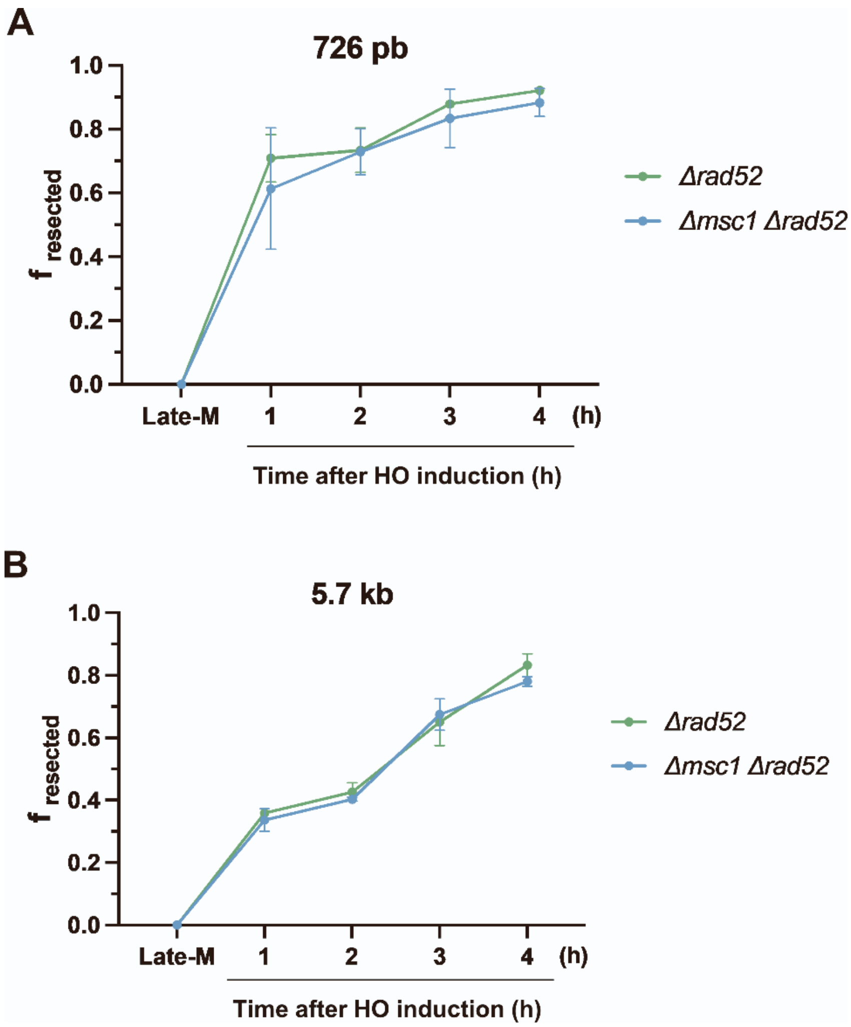

Figure S10, related to Figure 3. Late mitotic resection of the HO-mediated DSB ends in the Δ*rad52* and the Δ*rad52* Δ*msc1* strains. On the left, resection kinetics for an amplicon located 726 bp downstream of the HO-generated DSB (mean ± SEM, n=2). On the right, resection kinetics for an amplicon located 5.7 bp downstream of the HO-generated DSB (mean ± SEM, n=2); f resected is the fraction of resected DNA.

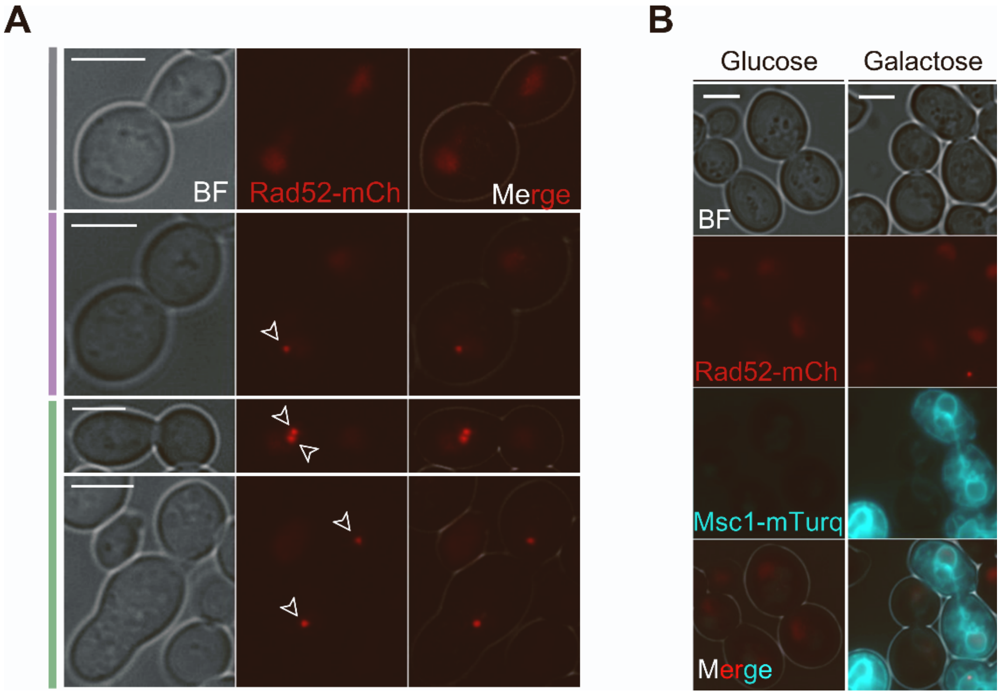

Figure S11, related to Figure 4. General pattern of Rad52 foci in late-M cells after DSBs. (A) Representative micrographs of late-M cells for each of the categories quantified in Figure 4A. Micrographs in the same color pattern (shown as a vertical line on the left). White arrows point to Rad52 foci. **(B)** Representative micrographs of late-M cells with (galactose) or without (glucose) Msc1-mTurquoise2. The examples correspond to the mock subcultures in Figure 4B, 2 h after carbon source shift. Scale bars correspond to 5 µm. BF, bright field.

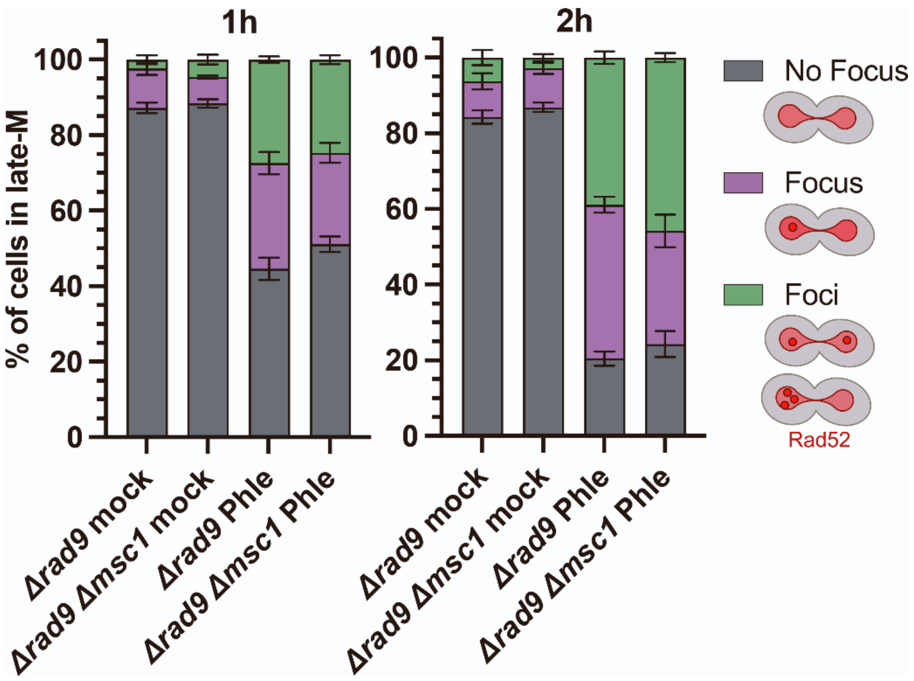

Figure S12, related to Figure 4. Rad52 foci after DSBs in the Δ*rad9* and Δ*rad9* Δ*msc1* strains. The experiment and the quantification as that shown in Figure 4A. Only phleomycin was tested here.

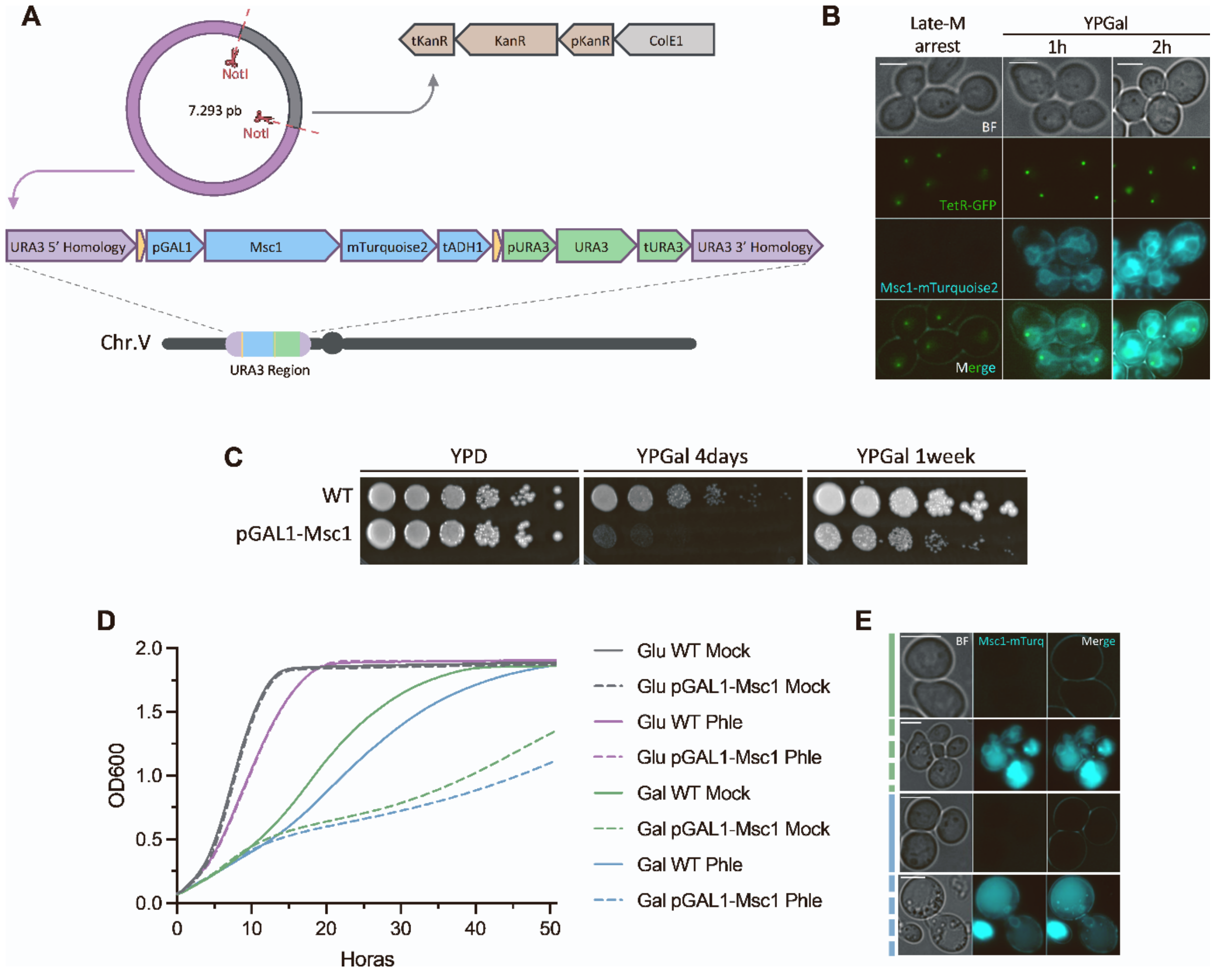

Figure S13, related to Figure 4. Short- and long-term Msc1 overexpression profiles. (A) Schematics of the integrative plasmid created by modular cloning for overexpressing *MSC1* under the *GAL* promoter. The construct is designed to be integrated ectopically at the *URA3* locus. Msc1 includes the mTurquoise2 tag at the C-terminus to verify expression by microscopy. Another variant of this plasmid was also created with the only difference that their integration is at the *HO* locus and has *LEU2* as marker. **(B)** Confirmation that Msc1-mTurquoise2 is overexpressed in a late-M arrest after a short incubation in YP galactose. Msc1-mTurquoise2 was clearly detected with less exposure time and intensity from the fluorescence source than the endogenous Msc1-eYFP after just 1h incubation; after 2h, signal saturation was common. Overexpressed Msc1 was located at other peripheral structures (ER and/or plasma membrane) aside from the NE. **(C)** Spot assay of long-term Msc1 overexpression. Parental (WT) and the *pGAL-MSC1* strains were grown overnight in YPD to log phase, the OD_{600} was adjusted to 0.5, 1:5 serial dilutions were made and then spotted onto the corresponding plates. The YPGal plate was photographed after 4 and 7 days. Note that Msc1 overexpression is toxic. **(D)** Growth curves of the WT and *pGAL-MSC1* strains under different inducing conditions (Glu or Gal) and with (Phle) or without (mock) concomitant moderate DNA damage caused by phleomycin (2 $\mu g \cdot mL^{-1}$). The initial inoculum for both strains was set to OD_{600}=0.05. Data are the mean of two independent experiments (the SEM is not represented but was less than 0.1 throughout). Note that both strains grow to

the same extent with glucose as the carbon source, regardless of whether phleomycin is added or not. With galactose as the carbon source, the growth of the strain with galactose-dependent Msc1 overexpression is clearly slowed. Msc1 overexpression neither increases nor suppresses Phle sensitivity. **(E)** Microscopy of cells taken from the growth curves in YPGal after 50h. In WT there is no signal in the blue channel, while in strains with the *pGAL1-MSC1-mTurquoise2* system a strong yet mislocated Msc1 signal was observed.

Table S1, related to Figure 1. Genes that encode proteins that significantly change their levels upon DSBs in late-M but not in G2/M.

HO-mediated DSBs		
Up-regulated		Down-regulated
YNL333W (*SNZ2*)<br>*YFL059W (SNZ3)*<br>***YLR178C (TFS1)****<br>***YPR160W (GPH1)****<br>***YGR256W (GND2)****<br>*YOL036W*<br>***YPR184W (GDB1)****	*YDR221W (GTB1)*<br>*YPL214C (THI6)*<br>*YGR281W (YOR1)*<br>***YML128C (MSC1)****<br>*YPR172W*<br>*YMR271C (URA10)*	*YHR083W (SAM35)*<br>*YLR417W (VPS36)*<br>*YDR440W (DOT1)*<br>***YEL046C (GLY1)****<br>*YKL082C (RRP14)*<br>*YKR020W (VPS51)*
Phleomycin-mediated DSBs		
Up-regulated		Down-regulated
YKL107W<br>*YMR105C (PGM2)*<br>***YGR256W (GND2)****<br>*YMR196W*<br>***YLR178C (TFS1)****<br>***YML128C (MSC1)****<br>*YLR001C*<br>***YPR184W (GDB1)****<br>*YIL136W (OM45)*<br>*YLR258W (GSY2)*<br>*YGR043C (NQM1)*<br>*YBR072W (HSP26)*<br>*YBR234C (ARC40)*	*YNR034W-A (EGO4)*<br>*YOR173W (DCS2)*<br>*YDL204W (RTN2)*<br>*YFL014W (HSP12)*<br>*YEL039C (CYC7)*<br>*YDR345C (HXT3)*<br>*YFL011W (HXT10)*<br>*YOL156W (HXT11)*<br>*YJL219W (HXT9)*<br>*YGR248W (SOL4)*<br>***YPR160W (GPH1)****<br>*YOR120W (GCY1)*	*YBR014C (GRX7)*<br>***YEL046C (GLY1)****<br>*YDR246W (TRS23)*<br>*YGR283C (UPA1)*<br>*YER126C (NSA2)*<br>*YLR056W (ERG3)*

*Genes whose products change significantly against both HO-mediated and phleomycin-mediated DNA damage are highlighted in bold type.

Table S2, related to Figure 1. Changes of abundance (log2) of known DSB signaling and repair proteins upon DSBs in G2/M and late-M.

Protein[1]	G2/M with HO		G2/M with Phle		Late-M with HO		Late-M with Phle	
	$-\log_{10}$ (p-value)	$\log_2$ (fold change)	$-\log_{10}$ (p-value)	$\log_2$ (fold change)	$-\log_{10}$ (p-value)	$\log_2$ (fold change)	$-\log_{10}$ (p-value)	$\log_2$ (fold change)
Chk1	0.47	-0.17	0.10	-0.34	1.33	-0.35	0.53	-0.12
Dcc1	-	-	-	-	0.18	0.02	1.08	0.51
Ddc1	-	-	-	-	-	-	-	-
Ddc2	0.56	-0.14	-	-	0.15	-0.08	0.20	-0.12
Dna2	-	-	2.03	0.25	-	-	-	-
Dnl4	-	-	-	-	-	-	-	-
Exo1	-	-	-	-	-	-	-	-
Mec1	-	-	0.61	-0.77	-	-	0.07	-0.43
Mms4	-	-	-	-	-	-	-	-
Mph1	0.29	0.01	-	-	-	-	-	-
Mre11	0.21	0.37	-	-	0.20	0.14	0.50	0.39
Msh2	0.10	0.08	0.27	-0.11	0.26	-0.21	0.06	-0.47
Mus81	-	-	-	-	-	-	-	-
Rad5	-	-	-	-	-	-	-	-
Rad9	-	-	-	-	-	-	-	-
Rad50	0.94	0.52	0.71	0.73	0.76	0.38	1.98	0.47
Rad51	0.86	0.31	1.34	0.62	0.02	-0.01	0.68	0.51
Rad52	0.44	0.15	0.27	0.15	0.11	-0.54	0.33	-0.19
Rad53	0.33	-0.01	-	-	0.15	0.24	0.06	0.13
Rad54	-	-	-	-	-	-	-	-
Rad55	-	-	-	-	-	-	-	-
Rad57	-	-	-	-	-	-	-	-
Rad59	-	-	-	-	-	-	1.09	-0.94
Rfa1	0.08	0.01	0.01	0.01	1.80	-0.39	2.91	-0.48
Rmi1	-	-	-	-	-	-	-	-
Sae2	-	-	-	-	-	-	-	-
Sgs1	-	-	-	-	-	-	-	-
Slx1	-	-	-	-	-	-	-	-
Slx4	-	-	-	-	-	-	-	-
Smc1	1.58	0.38	2.10	0.41	0.02	-0.01	0.46	0.27
Smc2	0.63	1.09	0.98	0.16	0.09	0.40	0.69	0.62
Smc3	1.95	0.63	1.07	0.32	0.19	-0.09	0.04	0.02
Smc4	0.52	0.51	0.14	0.31	0.76	0.47	0.62	0.22
Smc5	-	-	-	-	-	-	1.48	0.18
Smc6	-	-	-	-	-	-	-	-
Srs2	-	-	-	-	-	-	-	-

Tel1	-	-	-	-	-	-	-	-
Top3	-	-	-	-	-	-	-	-
Xrs2	-	-	-	-	-	-	-	-
Yen1	-	-	-	-	-	-	-	-
Yku70	-	-	-	-	-	-	-	-
Yku80	-	-	-	-	0.19	-0.06	0.29	0.17

[1] Proteins are ordered alphabetically. "-" indicates that the protein was not detected by the inclusion criteria set for the proteomics analysis (see STAR methods).

Table S3, related to Figure 4. Contingency table of patches of Msc1 vs. foci of Rad52 after 1h and 2h of phleomycin[1].

1h Phleomycin

Msc1 vs. Rad52	Rad52 foci	No Rad52 foci	Total
Msc1 Patch	63	59	122
No Msc1 patch	63	172	235
Total	126	231	357

2h Phleomycin

Msc1 vs. Rad52	Rad52 foci	NO Rad52 foci	Total
Msc1 Patch	90	55	145
NO Msc1 patch	65	107	172
Total	155	162	317

[1] Counted cells (N) came from a pool of three independent experiments as in Figure S1 (only DSBs by phleomycin). N>100 per experiment. Fisher's exact test p value < 0.0001 at both time points.

Table S4, related to STAR Methods. Strains used in this work.

Name	Genotype[1]	Origin	Use
FM2531	*MATa bar1Δ leu2-3,112 ura3-52 his3-Δ200 trp1-Δ63 ade2-1 lys2-801; cdc15-2:9myc::Hph; HMLα Δhmr::HIS3MX; leu2-3::LexA-TF-PlexOp:HO::LEU2*	Machín lab	Figs 1A; 2; 3A,B,C; S2A; S5; S6; S8 Table S1; S2
FM2790	FM2531; *MSC1:6HA::KanMX*	This study	Figs 1B,C
FM2787	*MATa bar1Δ leu2-3,112 ura3-52 his3-Δ200 trp1-Δ63 ade2-1 lys2-801; ade2-1::TetR:YFP::ADE2; tetO(5.6Kb)::1061Kb-ChrXII::HIS3; cdc15-2:9myc::Hph; MSC1:6HA::KanMX*	This study	Fig S2B
FM2831	FM2531; *MSC1:eYFP::KanMX*	This study	Figs 1D-G; S3B,C
FM3208	*MATa bar1Δ leu2-3,112 ura3-52 his3-Δ200 trp1-Δ63 ade2-1 lys2-801; NUP49:mCherry::NatNT2; HTA2:eCFP::TRP1; MSC1:eYFP::Hph*	This study	Fig S3A
FM2705	*MATa bar1Δ leu2-3,112 ura3-52 his3-Δ200 trp1-Δ63 ade2-1 lys2-801; ade2-1::TetR:YFP::ADE2; tetO(5.6Kb)::1061Kb-ChrXII::HIS3; cdc15-2:9myc::Hph; MSC1:mCherry::KanMX*	This study	Fig S3D
FM2823	*MATa bar1Δ leu2-3,112 ura3-52 his3-Δ200 trp1-Δ63 ade2-1 lys2-801; ade2-1::TetR:YFP::ADE2; tetO(5.6Kb)::194Kb-ChrXII::HIS3; cdc15-2:9myc::Hph; HTA2:mCherry::KanMX; MSC1:AID*:9myc::NatNT2; pADH1:OsTIR1:9myc::URA3*	This study	Fig S4
FM82	*MATa bar1Δ leu2-3,112 ura3-52 his3-Δ200 trp1-Δ63 ade2-1 lys2-801; Δrad52::kanMX4*	Machín lab	Figs 2A,B; S5A
FM2808	FM2531; *Δmsc1::NatNT2*	This study	Figs 2; 3A,B,C; S5; S6; S8
FM630	*MATa his3Δ1 leu2Δ0 met15Δ0 ura3Δ0 Δyap1::kanMX4*	Euroscarf	Fig S5B
FM2915	FM2808; *Δrad52::KanMX*	This study	Figs S9; S10

FM2917	FM2531; *Δrad52::KanMX*	This study	Figs S9; S10
FM2929	FM2531; *RAD52:mCherry::NatNT2*	This study	Figs 4A; S11A
FM2891	FM2808; *RAD52:mCherry::KanMX*	This study	Figs 4A; S11A
FM3071	FM2891; *NUP49:eGFP::TRP1; ura3-52::GAL1p:MSC1:mTurquoise2::URA3*	This study	Fig 4B; S11B
FM3228	FM2929; *Δrad9::UraMX4*	This study	Figs 3D,E; S12
FM3231	FM2891; *Δrad9::UraMX4*	This study	Figs 3D,E; S12
FM2948	FM2929; *NUP49:eCFP::TRP1*	This study	Fig 4C
FM2956	FM2891; *NUP49:eCFP::TRP1*	This study	Fig 4C
FM2878	FM2831; *RAD52:mCherry::NatNT2*	This study	Fig 4D,E; Table S3
FM3217	FM2531; *RFA1:eYFP::KanMX*	This study	Fig 4F
FM3223	FM2808; *RFA1:eYFP::KanMX*	This study	Fig 4F
FM2317	*MATa bar1Δ leu2-3,112 ura3-52 his3-Δ200 trp1-Δ63 ade2-1 lys2-801; ade2-1::TetR:YFP::ADE2; tetO(5.6Kb)::194Kb-ChrXII::HIS3; cdc15-2:9myc::Hph; CIN8:mCherry::KanMX6*	Machín lab	Fig S13
FM2817	FM2317; *ho::GAL1p:MSC1:Turquoise::LEU2::ho*	This study	Fig S13

[1] Semicolon (";") separates genetic modifications accomplished sequentially through transformation. Intermediate strains are omitted.
